# Macrophages amplify spontaneous activity of damaged sensory neurons in a human co-culture model of neuropathic pain

**DOI:** 10.1093/brain/awag088

**Published:** 2026-03-06

**Authors:** Paschalina Chrysostomidou, Zoe Hore, Domenico Somma, Julia Vlachaki Walker, Katy Diallo, Heather F. Titterton, Aziza Elmesmari, Lauramariú Schino, Siobhan Adesida, Mariola Kurowska-Stolarska, Franziska Denk, Greg A. Weir

**Affiliations:** 1 School of Psychology and Neuroscience, University of Glasgow, Glasgow G12 8QQ, UK; 2 Wolfson Sensory, Pain and Regeneration Centre (SPaRC), School of Neuroscience, King’s College London, London SE5 8AF, UK; 3 Institute of Immunity and Transplantation (IIT), Division of Infection and Immunity, University College London, London WC1E 6JF, UK; 4 Research into Inflammatory Arthritis Centre (RACE), School of Infection, Immunity and Inflammation, University of Glasgow, Glasgow G12 8QQ, UK

**Keywords:** iPSC technology, neuroimmune crosstalk, nerve injury, chronic pain

## Abstract

Neuropathic pain is a highly prevalent condition for which treatments are hampered by low efficacy and dose-limiting side effects. Injury to the somatosensory nervous system causes maladaptive plasticity that initiates and maintains chronic pain. Emerging evidence suggests that inflammatory cells of the innate immune system shape the response of the injured nervous system and thereby contribute to the pathogenesis of pain. Data from preclinical models and human patient biopsies have specifically implicated peripheral macrophage populations for a proalgesic role, yet how these cell types influence damaged sensory neurons and whether they directly contribute to neuronal hyperexcitability is unclear.

Here, we have developed an induced pluripotent stem cell (iPSC) co-culture system to study the interactions of macrophages and sensory neurons in a fully humanized experimental model. We found that analogous to endogenous counterparts, iPSC-derived macrophages (iMacs) display a dynamic molecular and functional profile that is highly dependent on neuronal state. Co-culture with injured iPSC-derived sensory neurons induces morphological, gene expression and secretory profile changes in iMacs that are consistent with the response of macrophages to nerve injury *in vivo*. iMacs in turn amplify spontaneous firing in damaged sensory neurons, implicating macrophages in this cardinal feature of neuropathic pain.

These results illustrate the utility of an iPSC-based model to study signalling between these two cell types; they support a role for macrophages in directly amplifying damaged sensory neuron activity and highlight disrupting pathological signalling between these cell types as a promising strategy for future analgesic drug development.

## Introduction

Neuropathic pain affects over 5 million people in the UK,^1^ most of whom are failed by current treatments and live with often disabling pain.^2^ Our knowledge of the underlying pathophysiology of pain progression has increased rapidly over recent years but this has so far failed to translate to major breakthroughs in treatment approaches. This suggests a need to expand the cell and signalling pathways traditionally targeted and to develop more advanced translational platforms in which new drug targets can be defined and tested.

Pain arises following damage because of maladaptive plasticity within the somatosensory nervous system.^3^ Excessive electrical activity of sensory neurons is key to the initiation and maintenance of neuropathic pain,^4^ and this has led to direct targeting of these neurons being the primary focus of analgesic development. However, a multitude of interacting cell types occupy the sensory nerve and dorsal root ganglion (DRG, where the cell bodies of sensory neurons are located) and contribute to nervous system plasticity following injury. Within this context, the precise environmental and cellular cues that drive sensory neuron hyperexcitability are not fully understood. Emerging evidence suggests that inflammatory cells of the innate immune system make an important contribution to neuropathic pain, but exactly which cell types are involved and the critical signalling pathways that they engage are still unclear. Bridging this gap in our knowledge is important as a better understanding may allow us to disrupt pathological signalling for therapeutic gain.

In animal models of peripheral nerve damage, macrophages at the site of injury and in the corresponding DRGs increase in number^5–7^ and undergo major changes in gene expression.^8–11^ These cells have been implicated in nerve regeneration^12,13^ and depletion studies have suggested that they initiate and maintain neuropathic pain behaviours.^5,6^ Macrophage populations have similarly been associated with the human condition, with macrophage-associated genes being found enriched in DRGs and nerve trunk tissue from patients with neuropathic pain.^14,15^ Using Morton’s neuroma as a model system for human neuropathic pain, a recent study found a positive correlation between greater macrophage presence and higher paroxysmal pain scores in patients and specifically identified an increased intraneural density of CD163^+^MARCO^+^ macrophages in neuroma tissue.^7^ However, despite the evidence implying a prominent role for macrophages in neuropathic pain, we do not yet have a good understanding of how macrophages and damaged sensory neurons interact, and crucially, whether macrophages directly contribute to neuronal hyperexcitability.

Our understanding of macrophage involvement in pain pathophysiology has been greatly advanced by the use of experimental animal models. However, most drug targets identified in these models have failed to translate to patient benefit, which could in part be due to differences between the species. This makes a compelling argument to diversify our experimental platforms and to establish relevant testing models based on human tissue. Here, we have developed a humanized co-culture system of sensory neurons (iSNs) and macrophages (iMacs) derived from induced pluripotent stem cells (iPSCs) and used this to model their interactions in the context of nerve injury. Our findings indicate that iSNs shape the morphology, gene expression and secretion of iMacs. This profile is altered to a putatively more pro-inflammatory state upon neuronal injury, and we identify features consistent with the response of macrophages to nerve injury *in vivo*. Finally, we show that iMacs are capable of directly augmenting damaged iSN hyperexcitability, thus replicating a cardinal feature of neuropathic pain. These findings demonstrate that disrupting pathological signalling between macrophages and damaged sensory neurons is a promising approach for future analgesic development. We therefore present our system which could be used as a translationally relevant analgesic discovery and testing platform.

## Materials and methods

### Induced pluripotent stem cell culture

Experiments were conducted with three iPSC lines that have been previously characterized and described: SBAD2 (derived from skin fibroblasts of a 51-year-old male^16^), SBAD3 (derived from skin fibro-blasts of a 36-year-old female^17^) and SFCi55ZsG (derived from skin fibroblasts of a female donor^18^). Induced PSCs were maintained and differentiated into iMacs and iSNs following established protocols,^17,19^ with only minor modifications, which are detailed in full in the Supplementary material. The iMacs/monocyte-derived macrophages (MoMs) were added to iSNs in a 1:1 ratio in N2/B27 Plus medium (Neurobasal Plus medium, 2% B27 Plus supplement, 1% N2 supplement, 1% Glutamax) supplemented with 25 ng/ml BDNF, GDNF, NT3 and NGF, and 10 ng/ml M-CSF. This ratio was chosen as while in rodents there are fewer, in human DRG, virtually every neuron is in contact with at least one macrophage,^20^ and many of these same neurons will also associate with additional macrophages along the nerve trunk and at peripheral terminals. Co-cultures were initiated when iSNs were mature (>45 days *in vitro*, DIV) and, in the case of our injury model, 18 h after iSN axotomy. 50% media changes were performed twice per week, and co-cultures were maintained for 1–42 days. Axotomy of iSNs was performed similarly to that previously described.^21^ Mature iSNs were manually lifted from their growth plates and dissected into smaller aggregates with a scalpel blade. Cell clusters were then mechanically dissociated to single cells by using fire-polished glass pipettes of decreasing bore size. The subsequent cell suspension was then replated onto Matrigel-coated glass and allowed to adhere for 18 h before addition of iMacs.

### Sample preparation for single-cell RNA sequencing of iMacs/MoMs

Cells from all conditions were generated, collected, processed and sequenced in parallel. On the day of isolation, cultures were enzymatically dissociated with 30 min of Accutase (ThermoFisher) followed by brief mechanical dissociation with a fire-polished pipette. Single cell suspensions were transferred to PBS containing 1:1000 eBioscience™ Fixable Viability Dye eFluor™ 780 (ThermoFisher, Cat. No. 65-0865-14) and incubated on ice for 20 min. Samples were maintained in dark conditions from this point onwards. Samples were centrifuged and washed in fluorescence-activated cell sorting (FACS) buffer containing PBS, 2% fetal bovine serum, 1% anti-anti and 0.5 mM EDTA (ThermoFisher, Cat. No. 15825388) before being resuspended in Human TrueStain FcX blocking buffer (BioLegend, Cat. No. 422301) diluted 1:10 in FACS buffer and incubated for 10 min on ice. Brilliant Violet 711 anti-human CD45 (BioLegend, Cat. No. 304049) (1:100) and a unique TotalSeq-A hashtag oligo (HTO) [A-0251 (BioLegend, Cat. No. 394601), A-0252 (BioLegend, Cat. No. 394603), A-0253 (BioLegend, Cat. No. 394605), A-0255 (BioLegend, Cat. No. 394609), A-0256 (BioLegend, Cat. No. 394611), A-0258 (BioLegend, Cat. No. 394615), A-0259 (BioLegend, Cat. No. 394617) and A-0260 (BioLegend, Cat. No. 394619)] were added directly to each tube and incubated for 30 min on ice. Samples were subsequently centrifuged, resuspended in FACS buffer and strained (100 μm strainer) into FACS tubes. Sorting was performed by a BD FACSAria III within the University of Glasgow Cellular Analysis Facility. The gating strategy is documented in Supplementary Fig. 4. Unstained cells, Viability Dye eFluor™ 780 stained cells and single stained control beads were used for compensation. Cells were sorted into 1.5 ml microcentrifuge tubes containing complete RPMI media (see the ‘MoMs’ section). Samples were processed and analysed as detailed in the Supplementary material.

### Cytokine/chemokine release assays

A custom U-PLEX assay platform (Meso Scale Discovery) was used as per the manufacturer’s instructions to profile the abundance of 14 cytokines/chemokines in the supernatant of cultures containing either iMacs only, iMacs with healthy iSNs, iMacs with damaged iSNs, healthy iSNs only or damaged iSNs only. Supernatant was taken after 3 days of culture and centrifuged to remove debris, before profiling using the U-Plex assay. Plates were read by a MESO SECTOR S 600 Reader. A standard curve was derived from signals from standards and generated with a four-parameter logistic model. Sample concentrations were determined by backfitting to the standard curve and multiplied by the dilution factor. Galectin-3 (ThermoFisher, Cat. No. BMS279-4), CCL3 (ThermoFisher, Cat. No. 88-7035-22) and CCL4 (ThermoFisher, Cat. No. 88-7034-88) were profiled using human ELISA kits according to the manufacturer’s instructions. Signal intensity was determined using a Multiskan Spectrum plate reader (ThermoFisher) and sample concentration determined using a standard curve.

### Immunocytochemistry

Macrophage-like cells and iSNs, both co-cultured and alone, were fixed either directly with 4% paraformaldehyde (PFA) for 15 min at room temperature, or by overlaying culture media with 4% PFA containing 10% sucrose for 1 h at room temperature. Coverslips were washed three times with PBS before incubating with primary anti-bodies diluted in PBS with 0.3% Triton™ X-100 (ThermoFisher, Cat. No. A16046.AE) and 10% normal donkey serum for 2 h at room temperature. After three PBS washes, cells were incubated with secondary antibodies for 2 h at room temperature before being stained with DAPI (Sigma, Cat. No. D9542) for 10 min. Coverslips were then washed three times with PBS, mounted onto Vectashield antifade medium (Vector Laboratories, Cat. No. H-1400-10) and allowed to harden for 1 h at room temperature before imaging. Primary antibodies used in this study: *α*-NF200 (1:1000, mouse, Sigma-Aldrich Cat. No. 266004), *α*-BRN3A (1:100, rabbit, Merck Cat. No. AB5945), IBA1 (1:500, guinea pig, Synaptic systems Cat. No. 234 308), *α*-GFP (1:1000, chicken, Abcam Cat. No. ab13970), *α*-ATF3 (1:500, rabbit, Novus Biologicals Cat. No. NBP1-85816), *α*-FABP5 (1:200, rabbit, ProteinTech Cat. No. 12348-1-AP) and *α-β*-tubulin III (1:1000, mouse, Sigma-Aldrich Cat. No. N5389). A range of species-specific secondary antibodies were used that were conjugated to Pacific Blue, Alexa fluor 488, Rhodamine Red-X, Alexa fluor 647 (Jackson ImmunoResearch) and Alexa fluor 488+ (ThermoFisher Scientific).

### Image acquisition and analysis

All fluorescence images were acquired with a Zeiss LSM900 Airyscan confocal microscope equipped with 405, 488, 561 and 640 nm diode lasers and analyses were performed using FIJI.

There were some scenarios where blinding the experimenter to the experimental group was not possible owing to the stark difference in culture appearance (e.g. iMacs alone versus co-culture, or iSNs with or without iMacs). In these instances, every effort was made to blind the experimenter to the signal being studied, as per the following approaches.

Quantification of ATF3 signal: iMac nuclei from cells cultured with healthy or damaged iSNs were identified by IBA1 and DAPI and labelled for region of interest (ROI) analysis, blind to ATF3 signal. Nuclei masks were then overlayed onto the ATF3 channel, and the mean grey area of each ROI was automatically calculated in FIJI. Values were aggregated across cells from multiple fields of view to derive an average ATF3 signal intensity per sample.

Quantification of FABP5 signal: iMac cell bodies from cells cultured with healthy or damaged iSNs were identified by IBA1 and labelled for ROI analysis, blind to FABP5 signal. Cell masks were then overlayed onto the FABP5 channel, and the mean grey area of each ROI was automatically calculated in FIJI. Values were aggregated across cells from multiple fields of view to derive an average FABP5 signal intensity per sample.

To mitigate confounds of batch processing for both ATF3 and FABP5 quantification, culture, fixation, immunocytochemistry (ICC) and image acquisition were run in parallel for each group from a given differentiation. For iMac morphology, ROIs were selected based on areas of dense iSN cell bodies, as opposed to IBA1 signal. A cell was classified as positive for secondary branching if any processes were observed extending from primary processes. For cell size analysis, IBA1 positive cells were outlined using FIJI and identified using the processing steps: (i) threshold-despeckle; (ii) make binary-convert to mask; (iii) fill holes; and (iv) wand selection. Any cell which was not accurately outlined using this automated procedure was manually traced. ROIs were subsequently quantified for their area.

### Whole-cell patch clamp electrophysiology

Whole-cell patch clamp recordings of iSNs were performed at room temperature (22°C) with a Multiclamp 700B amplifier and Digidata 1550 acquisition system (Molecular Devices). Data were low pass filtered at 2 kHz and sampled at 10 kHz and recordings were analysed by Clampfit 10 software (Molecular Devices). Filamental borosilicate glass capillaries (1.5 mm outer diameter, 0.84 mm inner diameter; World Precision Instruments) were pulled to form patch pipettes of 3–7 MΩ tip resistance and filled with internal solution containing (mM): 130 K-gluconate, 10 KCl, 2 MgCl_2_, 2 ATP-Na_2,_ 0.5 GTP-Na, 10 HEPES and 0.5 EGTA; pH was adjusted to 7.3 with KOH and osmolarity was set to 305 mOsm. Extracellular solution contained (mM): 140 NaCl, 3 KCl, 2 MgCl_2_, 2 CaCl_2_, 10 HEPES and 10 glucose; pH was adjusted to 7.3 with NaOH and osmolarity was set to 315 mOsm. Resting membrane potential values were corrected offline for a liquid junction potential of −15.5 mV. One minute following establishing whole cell access, a 2-min recording was made in bridge mode to derive resting membrane potential and spontaneous action potential firing. Input resistance (*R*_Input_) was derived from the membrane potential deflection caused by a 20 pA hyperpolarizing current pulse at −60 mV. To determine the minimal current injection to fire an action potential (rheobase), cells were depolarized from a holding potential of −60 mV by current steps (50 ms) of increasing magnitude (Δ10 pA) until an action potential was generated. Recordings were discarded in cases of poor whole-cell breakthrough (evident from excessive leak current or recording instability), or if neurons failed to fire an action potential to any level of current.

### Animals

Male and female C57BL/6JolaHsd mice were purchased from Envigo at 7 weeks of age and tissue was used to generate the single-cell RNA sequencing (scRNA-seq) dataset of mouse myeloid cells. Sixteen-week-old male and female macrophage Fas-induced apoptosis (MAFIA) transgenic mice were used for immunohistochemistry to allow GFP labelling of Csf1r-expressing cells.^22^ All animals were housed in groups of up to five in individually ventilated cages in a 12 h light–dark cycle. All experiments were carried out in ac-cordance with the United Kingdom Home Office Legislation (Scientific Procedures Act, 1986) and were approved by the Home Office to be carried out at King’s College London and the University of Glasgow. Mice underwent sham surgery or one of three models of nerve damage: partial sciatic nerve ligation (PSNL), spared nerve injury (SNI) or sciatic nerve crush. Animals were not randomized, but rather age-matched littermates were divided across groups, and allocation was not concealed. No animals were excluded from analysis. Details of surgical procedures and subsequent sample collection, processing and analysis are described in the Supplementary material.

### Quantification and statistical analysis

Data are expressed throughout as mean ± standard error of the mean (SEM). Statistical testing was performed with GraphPad Prism, with significance for all experiments placed at *P* < 0.05. A Student’s *t*-test was used to compare the mean of two groups, and when data were not normally distributed a non-parametric test was applied (Mann–Whitney). Electrophysiological measures were assessed by two-way ANOVA analysis, testing for main effects of injury and iMac presence, and subsequent Tukey’s *post hoc* tests for pairwise comparisons. Sample sizes are detailed in each figure legend.

## Results

### Establishing a humanized co-culture model

**Figure 1:**
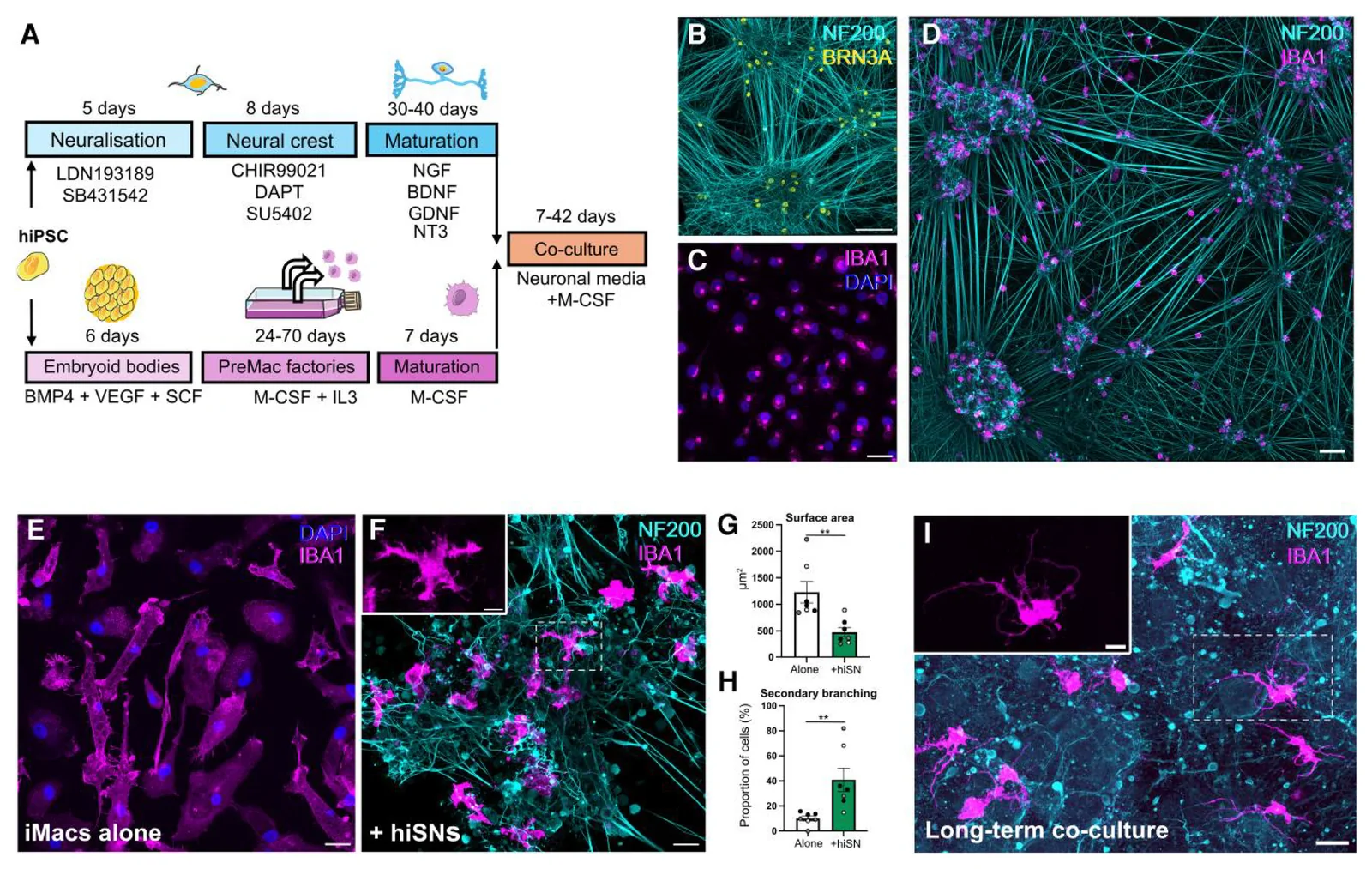
Co-cultures are viable and induce a more ramified iMac morphology. (**A**) Schematic overview of the experimental setup in which induced pluripotent stem cells (iPSCs) are differentiated in parallel to sensory neuron-like cells (iSNs)^19^ and macrophage-like cells (iMacs),^24^ before co-culture in defined media (see ‘Materials and methods section’). (**B**) Sensory neuron-like cells at DIV50 expressing the sensory neuron marker BRN3A, and exhibiting extensive neurite growth (NF200). Scale bar = 100 μm. (**C**) Macrophage-like cell precursors expressing the myeloid cell marker IBA1. (**D**) Representative image of iSNs and iMacs co-cultured for 14 days, illustrating the tendency of iMacs to associate with groups of iSN cell bodies. Scale bar = 100 μm. (**E**) Representative morphology of iMacs cultured for 7 days in the absence or (**F**) presence of healthy iSNs (hiSNs). (**G** and **H**) Co-culture reduces iMac cell size and results in a more ramified morphology. Data represent mean ± SEM, with values for individual differentiations also shown (*n* = 7, from three independent iPSC lines: black circles—SBAD2, grey circles—SBAD3 and white circles—ZsGreen). Mann–Whitney test (**G**) and two-tailed unpaired *t*-test (**H**). **P < 0.01. (**I**) Representative image of cells in co-culture for 42 days, showing that the two cell types co-exist for long periods and iMacs retain a ramified morphology. *Inset* scale bars = 10 μm and all other scale bars = 25 μm unless specified otherwise. DIV = days *in vitro*; hiPSC = healthy iPSC; SEM = standard error of the mean.

With the goal of generating co-cultures of human sensory neurons and macrophages, we took advantage of previously characterized iPSC differentiation protocols. Human iPSCs can be differentiated into neurons with a transcriptional and functional profile consistent with those of human DRG neurons (iSNs) by using a cocktail of small molecule inhibitors (Fig. 1A).^19,23^ The precise sensory neuron subtype that they most closely resemble is unclear; however, they exhibit features of nociceptors^16,19^ and have proved a valuable model to investigate the physiology of human sensory neurons.^16,25–27^ We generated iSNs from one healthy control iPSC line (SBAD2).^16^ These robustly differentiated into a homogeneous culture of neurons with extensive neurite outgrowth, and expressed the sensory neuron marker BRN3A (Fig. 1B). Concomitantly, we used a well-characterized embryoid body-based protocol to generate primitive macrophages from iPSCs (iMacs) in a process reminiscent of yolk sac haematopoiesis.^17,24^ The iMacs were generated from three healthy control iPSC lines (SBAD2, SBAD3^16^ and a previously characterized ZsGreen knock-in line^18^). We confirmed successful iMac differentiation for all three lines based on morphology, expression of canonical genes such as IBA1, CD45 and CD64, and phagocytic competence (Fig. 1C and Supplementary Fig. 1A–E). Macrophages are highly plastic, and their profile is dependent on the tissue in which they reside; and iMacs are similarly able to acquire specific tissue-resident macro-phage phenotypes in co-culture environments.^17,28^ The iSNs and iMacs co-cultured in neuronal-based media with the addition of low levels of Macrophage Colony Stimulating Factor (M-CSF) were viable, and iMacs largely associated with clusters of iSN cell bodies (Fig. 1D). While iMacs cultured alone in neuronal media exhibited an elongated or amoeboid morphology (Fig. 1E), iMacs associated with iSN cell bodies took on a more complex morphology, including a reduced size with an increased number of processes (Fig. 1F–H), consistent with the ramified morphology of endoneurial macro-phages in the sciatic nerve.^29,30^ Co-culture successfully maintained the iMac populations for long periods (6 weeks), with iMac morphology appearing to increase in complexity at these time points (Fig. 1I). These data demonstrate the feasibility of co-culturing these two cell types and suggest a functional interaction between them that alters the iMac state.

### Neuron damage alters iMac morphology and secretory profile

**Figure 2:**
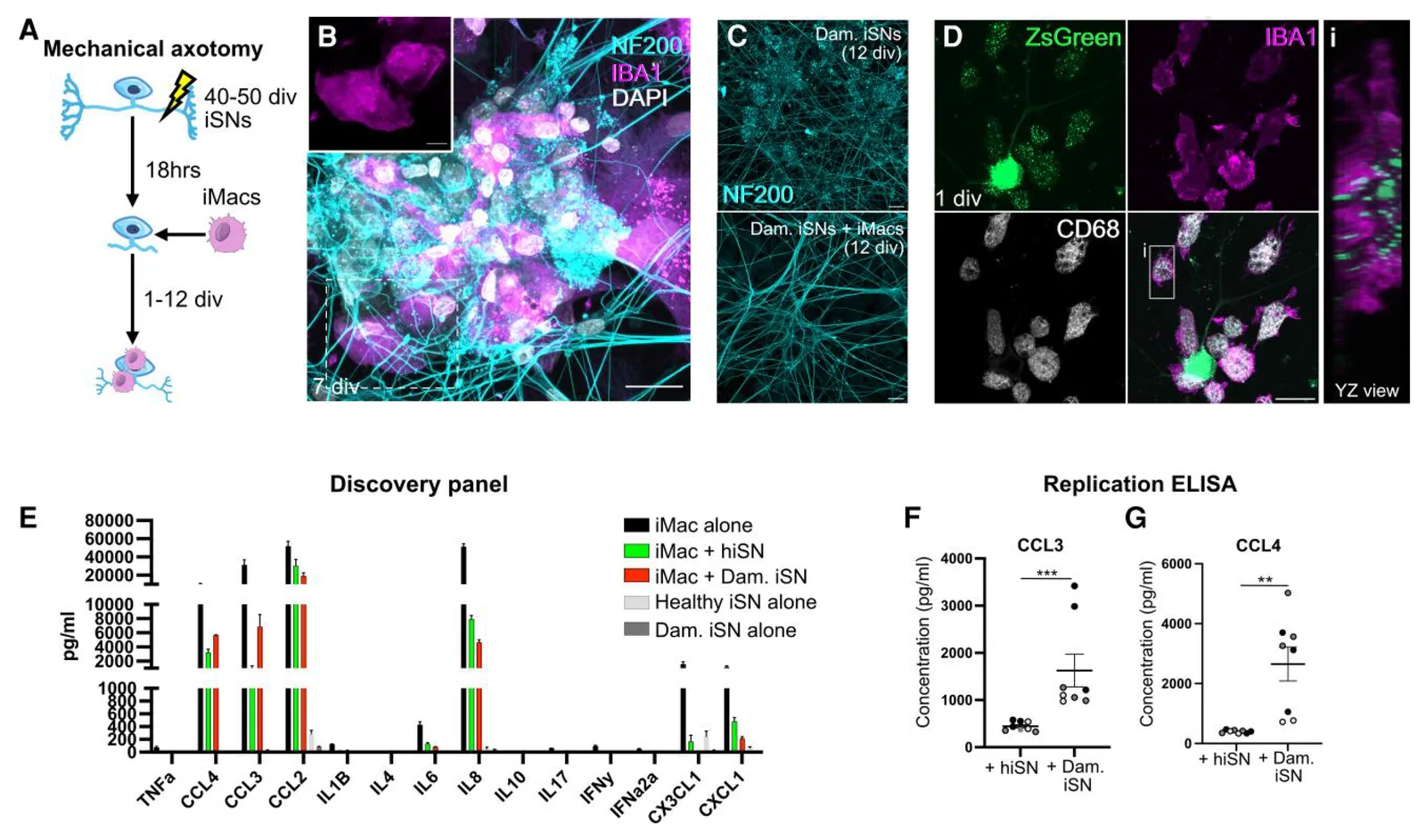
Mechanical injury to iSNs alters iMac morphology and secretory profile. (**A**) Schematic of the mechanical trauma model in which mature (DIV40–50) sensory neuron-like cells (iSNs) are mechanically dissociated and replated, resulting in axotomy. In co-culture experiments, macrophage-like cells (iMacs) are added to regenerating iSNs 18 h later. (**B**) Representative image of iMacs forming dense clusters with damaged iSN (Dam. iSN) cell bodies following 7 days of co-culture. Scale bar = 30 μm in main image and 10 μm in *inset*. (**C**) Sensory neuron-like cells 12 days after damage, cultured with and without iMacs. The addition of iMacs considerably reduces the amount of debris in damaged iSN cultures. Scale bars = 25 μm. (**D**) Image of damaged ZsGreen iSNs cultured with SBAD2 (non-fluorescent) iMacs for 24 h. CD68^+^ iMacs containing ZsGreen material can be seen surrounding a regenerating iSN. **D**(**i**) illustrates the highlighted region of interest as an orthogonal YZ view to observe ZsGreen material within the iMac cell body. (**E**) Discovery multiplex immunoassay detecting pain-relevant cytokines/chemokines in the supernatant 3 days following mono- or co-culture. Data represent mean ± SEM (*n* = 3, from SBAD2 and SBAD3 iPSC lines). (**F** and **G**) Individual CCL3 and CCL4 profiling in a verification cohort of supernatant samples taken following 3 days of co-culture. Data represent mean ± SEM, with values for individual differentiations also shown (*n* = 8, from three independent iPSC lines: black circles—SBAD2, grey circles—SBAD3 and white circles—ZsGreen). Two-tailed unpaired *t*-test (CCL4) and Mann–Whitney test (CCL3). **P < 0.01, ***P < 0.001. DIV = days *in vitro*; hiSN = healthy iSN; iPSC = induced pluripotent stem cell; SEM = standard error of the mean.

Following peripheral nerve damage *in vivo*, nerve and DRG-associated macrophages and microglia in the dorsal horn undergo major changes in morphology and gene expression.^8,10,31^ These changes are thought to be driven, at least in part, by soluble factors released directly by damaged sensory neurons, such as CSF1.^31,32^ To study how neuronal damage altered the interaction between our cells *in vitro*, we employed a mechanical trauma nerve injury model.^21^ Here, mature iSNs are dissociated and replated, resulting in complete axotomy (Fig. 2A). Damaged iSNs robustly express the canonical injury gene, ATF3, and rapidly regenerate neurites (Supplementary Fig. 2A), mirroring the regenerative capacity of the peripheral nervous system. *In vivo*, resident macro-phages within the sciatic nerve are reported to enlarge, retract their processes and take on a more rounded morphology following nerve damage,^29,30,33^ whereas DRG-resident macrophages acquire a more ramified morphology.^34^ The iMacs co-cultured with damaged iSNs for 7 days primarily formed dense clusters around neuronal cell bodies (Fig. 2B and Supplementary Fig. 2B). The iMACs in these neuron soma-associated clusters appeared to exhibit a more amoeboid profile (Fig. 2B), although quantification of individual cell morphology was hindered by the density of clusters. The iMacs not associated with clusters remained amongst the regenerating neurites and typically were large, flat and highly vacuolated (Supplementary Fig. 2B). Both states are consistent with macrophage morphologies that are observed in mouse nerve following injury (Supplementary Fig. 3). One notable divergence from the *in vivo* scenario, where DRG and nerve macrophage numbers rapidly expand following nerve injury, is that we did not observe acute damaged iSN-induced iMac proliferation, as evidenced by no increase in the prevalence of Ki67^+^ iMacs at acute time points of co-culture (Supplementary Fig. 2C).

Our axotomy model resulted in a large amount of cellular debris, composed of severed neurites and dead neurons. The addition of iMacs to cultures of damaged iSNs starkly reduced the amount of this debris (Fig. 2C), presumably via phagocytosis. To directly test this, we damaged ZsGreen iSNs prior to co-culture with SBAD2 (non-fluorescent) iMacs. iMacs were strongly positive for CD68, a marker of the phagolysosome. Clear ZsGreen particles could be observed within iMac profiles from as early as 24 h after co-culture, suggesting that iMacs actively phagocytosed neuronal debris from the cultures (Fig. 2D).

Next, we asked whether the secretory profile of iMacs was also neuron-state dependent by performing a discovery (*n* = 3) multiplex immunoassay for 14 soluble factors with previous association to nerve injury and pain. Appreciable levels of several soluble mediators were detected in iMac-containing cultures but not in monocultures of either healthy or damaged iSNs (Fig. 2E), suggesting that the source of secretion was iMacs. Similar to previous reports,^17^ we found that iMacs cultured alone had a greater secretory profile than iMacs co-cultured with healthy or damaged iSNs (Fig. 2E). We analysed one exemplar mediator (CCL4) and found that neuron-conditioned media was sufficient to suppress secretion, suggesting that a soluble mediator released by neurons was responsible for the reduced secretory profile (Supplementary Fig. 4). Nevertheless, our primary objective was to define mediators with increased secretion comparing damaged and healthy iSN co-culture, and within our targeted panel, damage-associated iMacs secreted more CCL3 and CCL4. These increases were verified in single-target immunoassays performed on independent and larger sample sizes (Fig. 2F and G). Both chemokines contribute to neuropathic pain behaviours in mouse models of nerve injury.^35,36^ Taken together, these data suggest that co-culture with damaged iSNs drives a morphological and secretory iMac profile that is distinct from that directed by healthy iSNs.

### Neuron state shapes iMac transcriptional profiles

**Figure 3:**
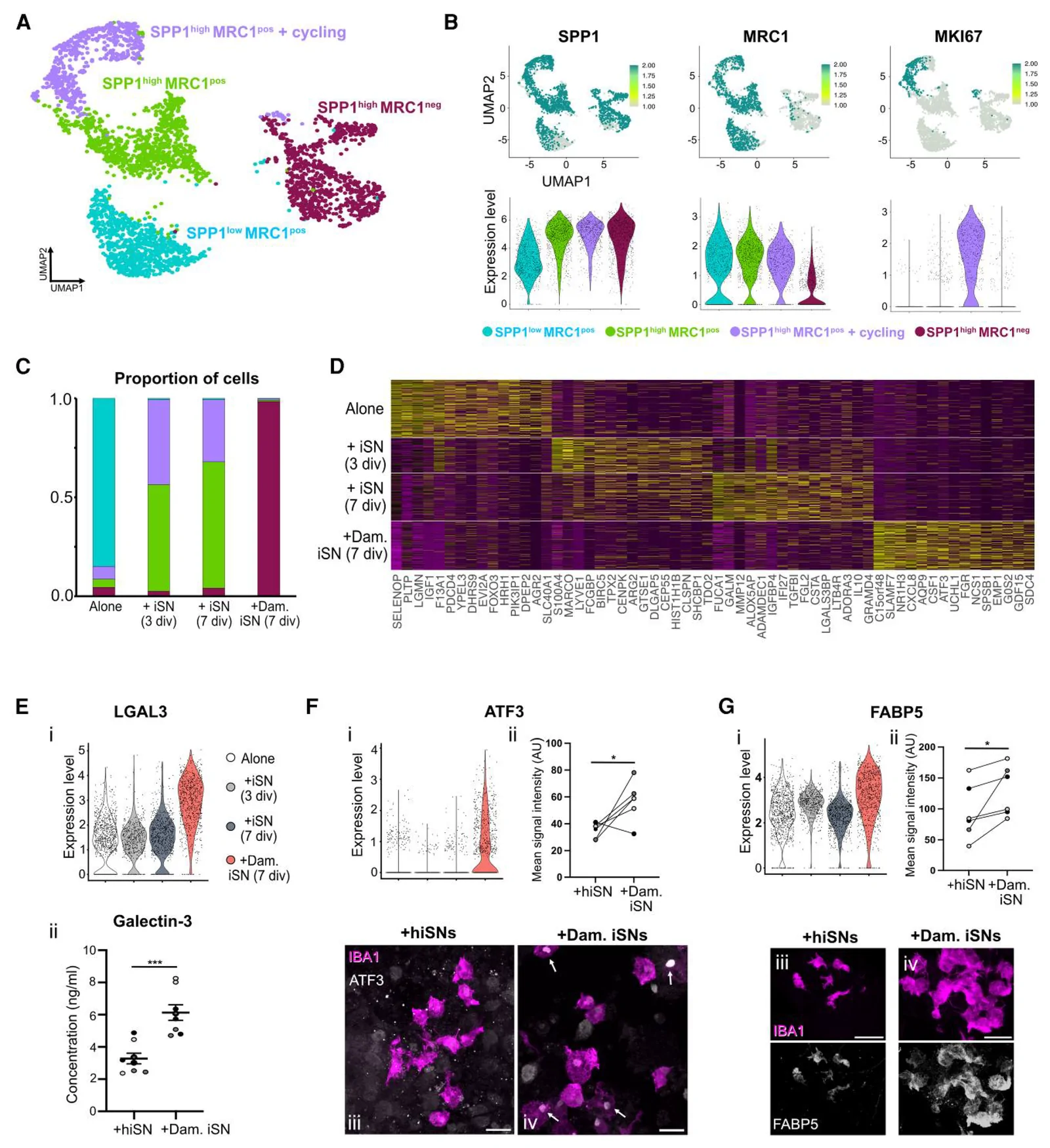
iMac gene expression profile is driven by co-culture condition. (**A**) UMAP plot of iMacs from all conditions showing four discrete clusters (*n* = 1 differentiation and one iPSC line): total cells (3157), cultured alone (987 cells), with healthy iSNs for 3 (576 cells) and 7 days (791 cells), and for 7 days with injured iSNs (803 cells). (**B**) UMAP feature plots and corresponding violin plots of the genes used to describe each cluster. Each dot is a cell that was sequenced; green dots indicate those positive for a given gene. (**C**) Proportion of iMacs from different conditions assigned to each of the four clusters. Note that most iMacs from each condition were assigned to one cluster (or two in the case of co-culture with healthy iSNs). (**D**) Heat map showing gene expression profile organized by iMac condition. The top 15 differentially expressed genes per condition are shown. [**E**(**i**)] Violin plot and validatory immunoassay (**ii**) for Galectin-3, showing its upregulation in iMacs co-cultured with damaged iSNs. The immunoassay was performed on supernatant from DIV7 co-cultures. Data represent mean ± SEM, with values for individual differentiations also shown (*n* = 8, from three independent iPSC lines: black circles—SBAD2, grey circles—SBAD3 and white circles—ZsGreen). Two-tailed unpaired *t*-test. ***P < 0.05. [**F**(**i**)] Violin plot showing upregulation of the injury gene ATF3 in iMacs co-cultured with injured iSNs. Quantification of mean ATF3 signal intensity (**ii**) and representative images of iMacs co-cultured with healthy (**iii**) or injured iSNs (**iv**) for 7 days, illustrating cells with nuclear ATF3 (white arrows). [**G**(**i**)] Violin plot for FABP5. Quantification of mean FABP5 signal intensity (**ii**) and representative images of iMacs co-cultured with healthy (**iii**) or injured iSNs (**iv**) for 7 days illustrating FABP5 expression. For quantification in **F** and **G**, paired values represent independent differentiations in which co-culture, ICC processing and image analysis were performed in parallel (*n* = 6, from three independent iPSC lines: black circles—SBAD2, grey circles—SBAD3 and white circles— ZsGreen). Two-tailed paired *t*-test. *P < 0.05. Scale bars = 25 μm. Dam. iSN = damaged iSN; DIV = days *in vitro*; hiSN = healthy iSN; iMACs = macrophage-like cells (iMacs); iPSC = induced pluripotent stem cell; iSNs = sensory neuron-like cells; SEM = standard error of the mean; UMAP = Uniform Manifold Approximation and Projection.

Macrophage gene expression is highly plastic and dependent on the local tissue environment and context (e.g. injury versus no injury). We performed a hypothesis-generating single-replicate experiment to explore the global gene expression profile of iMacs cultured under different conditions. SBAD2 iMacs were cultured alone, with healthy iSNs for 3 or 7 days, or cultured with damaged iSNs for 7 days. The iMac samples were subsequently purified from co-cultures by FACS based on viability and CD45 expression (Supplementary Fig. 5). Cells from each condition were labelled with an identifying hashtag antibody before being pooled into a single tube (to reduce sequencing variability) and scRNA-seq was then performed. Unbiased clustering separated iMacs into four discrete clusters marked by varying levels of SPP1, which encodes osteopontin, MRC1, which encodes the mannose receptor CD206, and MKI67, which marks actively cycling cells (Fig. 3A and B). Of note, SPP1 is upregulated in several disease-associated macrophage/microglial states, including CNS degeneration, ageing and injury,^37,38^ and MRC1 expression has been used to identify tissue resident macro-phages implicated in the resolution of pain following inflammation and surgical wound injury.^39,40^ Demultiplexing of the cells based on the hashtag identifier revealed that molecular clusters were broadly represented by discrete culture conditions. The iMACs cultured alone were classified as SPP1^low^MRC1^pos^, while iMacs cultured with healthy iSNs were represented in SPP1^high^MRC1^pos^ cycling and non-cycling populations. The iMACs cultured with damaged iSNs were found to be SPP1^high^MRC1^neg^ (Fig. 3C). In addition to these marker genes, cells from each condition could be defined by distinct patterns of gene expression (Fig. 3D). We explored whether iMac gene expression was dependent on iSN state (healthy versus damaged) and performed hypothesis-generating (owing to the single biological replicate) pseudo-bulk analysis to compare the two conditions. The iMacs cultured with damaged iSNs were enriched for expression of several genes that characterize a neuro-degenerative disease-associated microglial (DAM) state (such as ABCA1, LPL, CD9 and APOC1^41^) and those associated with proinflammatory conditions (such as C15orf48, CXCL8, NR1H3 and SLAMF7), while they exhibited a concomitant downregulation of genes associated with alternatively activated macrophage populations (such as DAB2, STAB1 and MMP12^42–44^; Supplementary Table 1). Several of the genes enriched in iMacs co-cultured with damaged iSNs have previously been implicated in neuropathic pain. We chose to validate increased expression of three of these genes that are known to play a role in macrophage biology: LGAL3 (encoding galectin-3), ATF3 and FABP5.^45–50^ The iMacs secreted greater levels of galectin-3 and exhibited higher protein expression of ATF3 and FABP5 when co-cultured with damaged versus healthy iSNs, across biological replicates (SBAD2, SBAD3 and ZsGreen derived; Fig. 3E–G).

Macrophages can be characterized as tissue-resident or infiltrating. Following peripheral nerve injury, the site of the lesion is characterized by extensive monocyte infiltration and proliferation of resident macrophage populations; moreover, DRG-resident macrophages have also been shown to proliferate, albeit to a lesser extent.^8,10,11,51,52^ While we used iMacs, which more closely resemble tissue-resident macrophages for our primary model, we sought to test whether MoMs responded similarly to co-culture with iSNs. MoMs derived from a healthy female donor were successfully maintained in co-culture with iSNs (Supplementary Fig. 6A). Similar to iMacs, MoMs altered their gene expression dependent on the presence and state of iSNs (Supplementary Fig. 6B–E and Supplementary Table 2). While clusters were described by different molecular markers, many of the genes enriched in MoMs co-cultured with damaged iSNs were also found to be enriched in iMacs under similar conditions (Supplementary Fig. 6F and Supplementary Table 3). ATF3 and LGAL3, but not FAPB5, were found enriched in the damaged neuron condition (Supplementary Fig. 6G). Taken together, these data illustrate that iMac gene expression is highly plastic and dependent on neuronal context, with iMacs cultured with damaged iSNs taking on a putatively more pro-inflammatory profile, features of which are shared with MoMs.

### Validation with *in vivo* murine datasets

**Figure 4:**
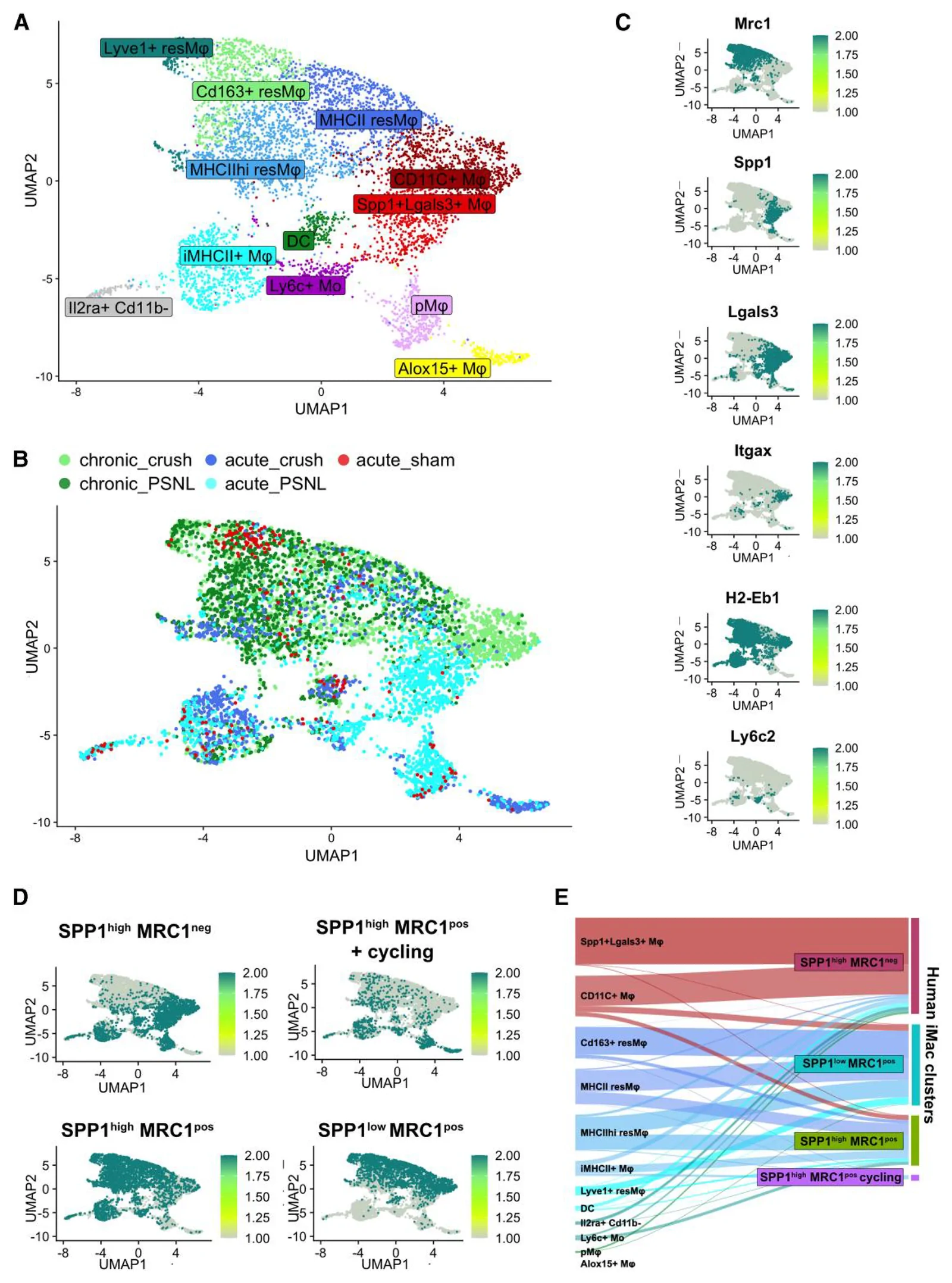
Mouse scRNA-seq data reveal that Spp1+ and Mrc1 macrophages are also a feature of nerve injury *in vivo*. (**A**) UMAP plot of CD11b^+^ myeloid cells derived from mouse nerve after injury at acute (1 week) and chronic (15 weeks) time points. (**B**) Injury state superimposed on the UMAP space. Each dot is a cell and the colour of each dot denotes which experimental group the cell derived from: 1 week (acute) post-sham, or 1 week (acute) or 15 weeks (chronic) post-partial sciatic nerve ligation or sciatic nerve crush injury. (**C**) Gene expression of several marker genes plotted onto the UMAP clusters. Each dot is a cell that was sequenced; green dots indicate those positive for a given gene. (**D**) Marker genes for the iMac clusters projected onto the mouse scRNA-seq UMAP space using the UCell package. To be included in the analysis, marker genes had to be expressed in at least 10% of cells in each cluster and had to differ in their expression from the other clusters by at least 20% points at adjusted *P* < 0.05. Injury-induced SPP1^high^MRC1^neg^ iMacs map well onto mouse injury-induced macrophages (majority blue clusters in **B**), while SPP1^low^MRC1^pos^ and SPP1^high^MRC1^pos^ map more prominently onto mouse resident (res) macrophage clusters labelled in **A**. (**E**) Sankey plot showing how mouse macrophage scRNA-seq clusters obtained from the sciatic nerve after injury map onto the clusters observed in our human iMac clusters. Mouse macrophage clusters on the *left*, human macro-phage clusters on the *right*. Of all 6334 cells which were seeded from the human data, 65% could not be assigned and are not included here; it is unclear to what extent this is due to true biological differences or due to technical challenges associated with batch variability and orthologue conversion. iMac = macrophage-like cell; scRNA-seq = single-cell RNA sequencing; UMAP = Uniform Manifold Approximation and Projection.

While there is evidence that macrophage populations are involved in pain progression across different types of nerve injury,^6,53,54^ their role is likely context-dependent and may evolve during the transition from initiation to maintenance of neuropathic pain. To better understand whether our *in vitro* system recapitulates features of the *in vivo* scenario, we profiled macrophage populations in the sciatic nerve of mice following sham surgery, as well as at early (1 week) and late (15 weeks) time points following two types of mechanical nerve trauma: partial sciatic nerve ligation (PSNL), which results in a non-resolving injury, and nerve crush, which allows nerve regeneration and recovery. We collected live single myeloid cells based on CD45^+^/CD11b^+^ staining (excluding Ly6G^+^ neutrophils at acute time points) (Supplementary Figs 7 and 8) from the sciatic nerve of sham and nerve-injured animals. Cells from different conditions were labelled with hashtag oligos and combined as described in the ‘Materials and methods’ section. Consistent with our *in vitro* data describing naïve clusters with MRC1, Mrc1^+^ resident macrophage clusters, characterized by expression of Cd163, Lyve1 and MHCII were the predominant populations present following sham surgery. At the acute time point, PSNL was characterized by an enrichment of an Spp1^+^Lgals3^+^ cluster and the presence of putative phagocytic cells (pM*φ*), whereas induced MHCII macrophages (iMHCII^+^ M*φ*) predominated in the crush, alongside a clear enrichment for Alox15^+^ putative wound-healing macrophages (likely reflecting the favourable regenerative conditions of the crush model) (Fig. 4A–C and Supplementary Fig. 9A). With both injury states, we also detected an expansion of Spp1^+^ CD11c^+^ macro-phages, which resemble injury-associated microglia described in the CNS.^37^ Finally, we detected small numbers of putative dendritic cells (DC), non-myeloid cells that escaped our sorting strategy (Il2ra+) and Lyc6+ monocytes. The last cell type likely appears rarer than it is *in vivo*, because monocytes die preferentially during scRNA-seq workflows. At the chronic time point, the differences between injury states appear to subside, with resident populations once more predominating across most samples.

We sought to assess the similarity of our human macrophage populations to their mouse counterparts. Consistent with the human *in vitro* data, Spp1 expression was enriched in mouse injury-induced macrophage clusters, whereas, conversely, Mrc1 was enriched in resident macrophage populations that were abundant in the uninjured state (Fig. 4C). We projected marker genes from our human clusters onto the mouse scRNA-seq Uniform Manifold Approximation and Projection (UMAP) space using the UCell package. Injury-induced SPP1^high^MRC1^neg^ iMacs mapped well onto mouse clusters that were enriched at acute time points following PSNL and crush, while healthy iSN-associated SPP1^high^MRC1^pos^ iMacs mapped more prominently onto mouse naïve-state resident macrophage clusters (Fig. 4D). Global expression profile alignment illustrated that injury-enriched mouse clusters mostly mapped to injury-induced SPP1^high^MRC1^neg^ iMacs, while resident macrophage populations map to both iMacs cultured alone (SPP1^low^MRC1^pos^) and iMacs cultured with healthy iSNs (SPP1^high^MRC1^pos^). Finally, we observed enrichment of our three neuropathic pain-associated genes (Lgals3, Atf3 and Fabp5) across murine injury states (Supplementary Fig. 9B).

Macrophages in the nerve and DRG diverge in their molecular response to nerve injury.^11^ While we did observe some important DEGs in common, we found limited overall overlap between our injury-induced SPP1^high^MRC1^neg^ iMac cluster and two previously published datasets of mouse DRG-resident macrophages following acute nerve injury (Supplementary Fig. 10A).^11,55^ In a final analysis, injury-induced SPP1^high^MRC1^neg^ iMacs were enriched for genes associated with a recently described macrophage subpopulation, Macro18, a nerve-resident macrophage population prevalent in sensory nerves from patients with polyneuropathy (Supplementary Fig. 10B).^56^

These data illustrate the heterogeneity and dynamic nature of the macrophage response to nerve injury *in vivo*. They also demonstrate shared features between our injury-induced iMac populations and macrophage clusters observed at acute time points following nerve injury *in vivo*, supporting the validity of the *in vitro* model.

### iMacs augment hyperexcitability associated with neuron damage

**Figure 5:**
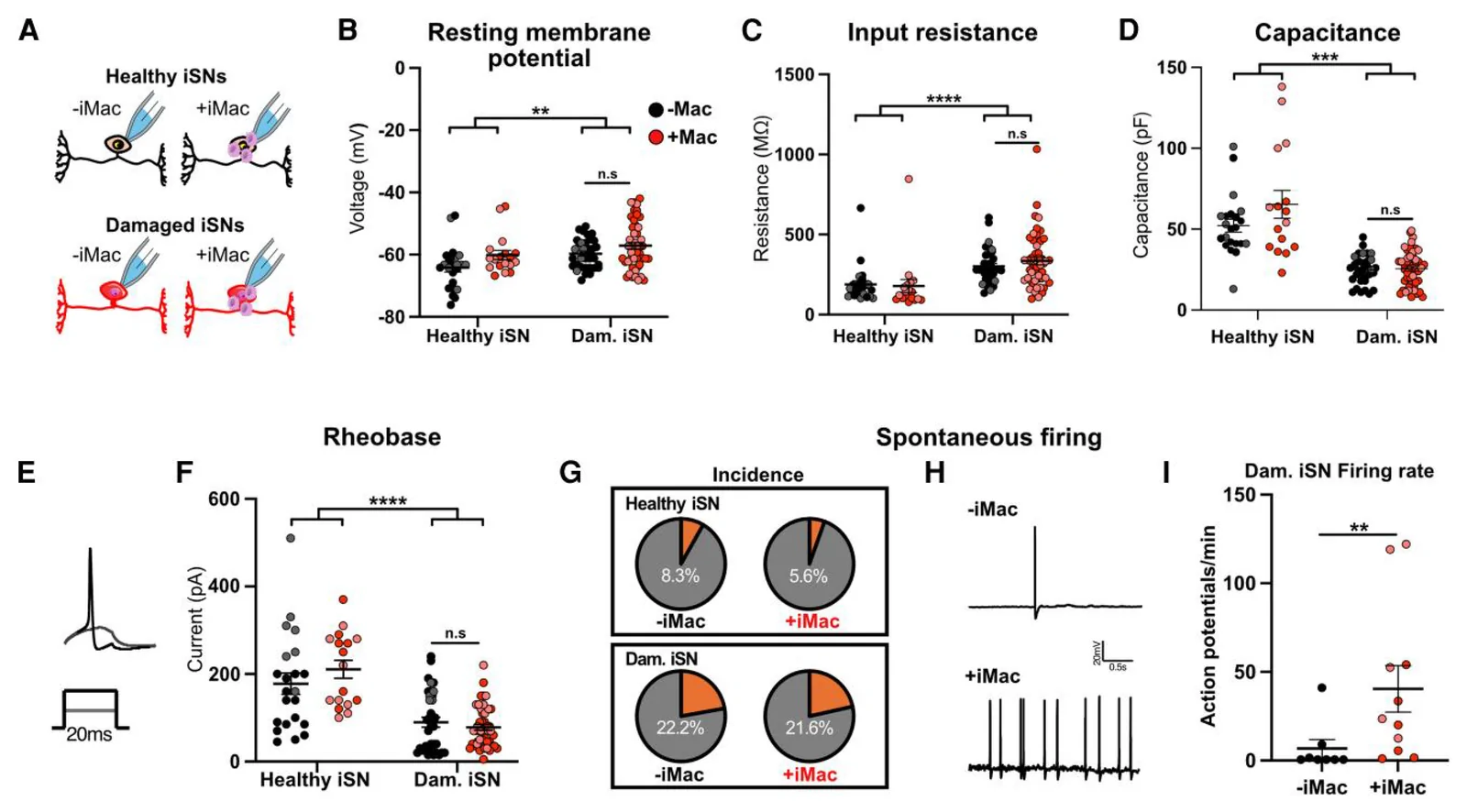
iMacs augment iSN spontaneous activity following injury. (**A**) Patch clamp recordings were taken from healthy sensory neuron-like cells (hiSNs) and injured iSNs 7 days following injury, with or without iMac co-culture. (**B** and **D**) Passive membrane properties of iSNs across the conditions. Significance is shown for a two-way ANOVA analysis testing for main effects of injury and iMac presence and subsequent Tukey’s *post hoc* tests (full numerical details of all tests are provided in Supplementary Table 1). (**E**) Representative image showing action potential firing of a healthy iSN to incremental current injections. (**F**) Mean ± SEM rheobase values. (**G**) Proportion of spontaneously active iSNs as defined by the firing of at least one action potential during a 2-min period. (**H**) Example traces of spontaneously active damaged iSNs without (*top*) and with (*bottom*) iMacs. (**I**) Action potential firing frequency of spontaneously active damaged iSNs (Dam. iSNs). Mann–Whitney test. Note, healthy iSN groups could not be included in this analysis owing to samples only including 1–2 spontaneously active neurons. All data represent mean ± SEM with values for individual iSNs also shown (derived from two independent iPSC lines: black/red circles—SBAD2, grey/light red circles—SBAD3). **P < 0.01, ***P < 0.001, ****P < 0.0001. iMac = macrophage-like cell; iPSC = induced pluripotent stem cell; SEM = standard error of the mean.

Hyperexcitability and ectopic activity of sensory neurons are fundamental drivers of neuropathic pain.^4,57,58^ Macrophages secrete many soluble mediators that can sensitize sensory neurons, but there is no causal evidence demonstrating that macrophages directly influence sensory neuron activity following damage. We performed patch clamp recordings to assess neuron injury-induced changes in excitability and whether the presence of iMacs further influenced neuronal activity (Fig. 5A). In terms of passive membrane properties, damaged iSNs exhibited a depolarized resting membrane potential, an increase in input resistance and a reduced capacitance (Fig. 5B–D). The latter likely reflects the loss of neurites and a shrinkage of soma size. Evoked action potential threshold (rheobase) was also reduced following neuronal damage (Fig. 5E and F); however, this result should be considered in the context of a reduced capacitance of these neurons. While these data provide evidence for hyperexcitability following damage, there were no differences in passive properties or evoked firing between injured iSN with or without iMacs (Fig. 5B–F). Uninjured nociceptors are usually electrically dormant in the absence of stimulation and even following nerve injury paradigms, spontaneous activity is only seen at low levels *in vitro*,^57^ which contrasts with *in vivo* recordings.^59^ *Ex vivo* mouse DRG neurons exhibiting spontaneous activity have greater input resistance than those that do not,^60^ and so we asked whether our injury paradigm induced changes in spontaneous activity. A greater proportion of iSNs exhibited spontaneous activity following injury (3 of 42 healthy iSNs versus 19 of 87 damaged iSNs, *P* = 0.046, Fisher’s exact test, with and without iMac groups combined), but there was no difference between conditions with or without iMacs (Fig. 5G). However, while incidence was no different, we observed a stark difference in the frequency of firing of those neurons exhibiting spontaneous activity. Damaged iSNs alone fired spontaneous action potentials (APs) at low frequency (6.8 ± 5.0 APs/min), whereas equivalent neurons co-cultured with iMacs exhibited firing at considerably increased frequency (40.5 ± 13.1 APs/min) (Fig. 5H and I). Together, these data suggest that neuronal damage induces hyperexcitability. They also show that spontaneous activity, a cardinal feature of neuropathic pain, can be directly augmented by macrophages.

To determine whether the action of iMacs required cell–cell contact, we performed further recordings from damaged iSNs in co-culture with iMacs. Neurons were visually inspected during patch clamp recordings and classified as having soma likely in direct contact (with at least one iMac), or devoid of iMac contact (Supplementary Fig. 11A). Note, this experimental setup does not test for the contribution of contact onto neurites. These data replicated the finding of high frequency of spontaneous firing in these cultures and found no difference between groups (Supplementary Fig. 11B), suggesting acute cell body contact is not required to mediate the sensitizing effect. To look for evidence of intercellular communication, we performed ligand–receptor analysis with a recently published dataset of healthy iSNs at different stages of maturity.^61^ Damaged iSN-associated SPP1^high^MRC1^neg^ iMacs had increased interactions compared to healthy iSN-associated SPP1^high^MRC1^pos^ iMacs, particularly for pathways associated with extracellular matrix signalling and cell adhesion (Supplementary Fig. 12A and B). SPP1^high^MRC1^neg^ iMacs had higher interaction scores for several receptors with known capacity to directly sensitize sensory neuron activity, including CD44, ALK, ITGB1/TGAA3 and NRP1^62–65^ (Supplementary Fig. 12B and C). CXCL8 (IL-8) has also been shown to heighten the excitability of sensory neurons,^66^ although which receptor this requires is not yet known. These data lay the ground-work for future investigations of candidate signalling pathways involved in iMac influence on iSN excitability.

## Discussion

Here we describe the first humanized model to study the interactions between sensory neurons and macrophages following neuron injury; we use this to reveal a hitherto postulated but never empirically demonstrated ability of macrophages to directly sensitize the electrical activity of damaged sensory neurons. Injury to human (*in vitro*) and mouse (*in vivo*) sensory neurons initiates a major switch in associated macrophage identity, with overlap in gene expression across the systems validating our *in vitro* model. These data demonstrate the opportunity to use co-cultures of iSNs and iMacs for discovery science and as the basis for future work developing this system into an analgesic testing platform, usable at scale.

### Validity of the human co-culture model

Our results illustrating the plasticity of iMacs *in vitro* mirrors what has been observed by others when co-culturing them with CNS neurons^17,28,41^ and the remarkable extent to which macrophage identity *in vivo* is dependent on the local tissue environment.^67^

The SPP1^high^MRC1^neg^ macrophage profile abundant in co-cultures with damaged iSNs generally mapped well onto mouse clusters that are enriched in the damaged sciatic nerve at early time points following injury *in vivo*. SPP1 is not a typical classifier of peripheral nerve-associated macrophages but is a common marker of microglia associated with brain injury and disease.^37,68^ We found Spp1 to be abundant in clusters enriched in our mouse samples of acute injury, consistent with results from previous studies.^9^ One aspect where our human and mouse cell types differed was the expression of SPP1 in iMacs co-cultured with uninjured neurons. In mice, Spp1 is not only associated with injury, but is also expressed by microglia close to emergent axon tracts during brain development.^37^ Sensory neuron-like cells exhibit features of a fully differentiated nociceptor at the time of our assays but are still maturing and in a period of neurite growth, providing a plausible explanation for why SPP1 expression was enriched in both conditions in our human system.

We found consistent upregulation of neuropathic pain-associated genes (ATF3, LGALS3 and FABP5) across *in vitro* and *in vivo* experimental platforms of nerve injury, although FABP5 was not enriched in MoMs. Study of ATF3 following nerve injury has largely focused on *de novo* neuronal expression, given the protein’s role in nerve regeneration.^69,70^ However, ATF3 has a well described role in macrophage biology where it is thought to act as a repressor of pro-inflammatory gene expression and cytokine release.^49,71^ LGALS3 encodes the multi-functional protein Galectin-3, which is a TREM2 ligand with described roles in several aspects of macrophage biology. It has been implicated in various neurological diseases, including neuropathic pain, where knockout or pharmacological inhibition of Galectin-3 reduces mouse pain behaviours following nerve injury.^46,72^ Beyond gene expression changes, we observed increased secretion of CCL3 and CCL4 from iMacs co-cultured with damaged iSNs. While the role of CCL2 as the primary monocyte/macrophage recruitment signal following peripheral nerve damage is well studied,^73,74^ the contribution of CCL3 and CCL4 is less so. Knockout of CCL3 or the major cognate receptor of CCL4 (CCR5) has no effect on macrophage recruitment to damaged nerves,^74,75^ yet signalling from both contributes to pain behaviours.^35,36^ It is therefore possible that these chemokines function to modify macrophage profile,^76,77^ or to directly sensitize sensory neurons.^78,79^ All of these factors have previously been proposed as viable therapeutic targets,^50,72,80^ and therefore our model system offers an attractive platform in which to test their contribution. However, there were several soluble mediators, which are reported to be produced by macrophages *in vivo* following nerve injury, that we did not observe in our culture system (e.g. TNF*α*, CCL2 and IL1*β*).^81^ Relatedly, we failed to see iMac proliferation in response to iSN damage. These features for which the *in vitro* system does not fully model biology as known *in vivo* may be due to damaged iSNs not releasing the full repertoire of damage-associated signals, or perhaps more likely could be caused by the absence in our culture system of other cell types important for driving the full macrophage response to nerve injury.

Few (if any) studies have profiled macrophage biology *in vivo* at chronic time points following different types of nerve injury. We observed that differences in macrophage clusters between resolving and persistent nerve damage appeared to subside over time, highlighting the evolving macrophage response. It is not clear how this relates to symptoms, with evidence suggesting that pain is not only limited to non-resolving nerve injuries, like PSNL, but can also arise from nerve crush,^82^ as well as during reinnervation.^83^ Mechanistically, it remains to be determined if and how the differences we observed in acute macrophage phenotypes influence other inflammatory and stromal cell types long-term.

### Macrophages directly amplify damaged sensory neuron ectopic activity

Sensory neurons undergo major transcriptional changes in response to injury *in vivo*.^84,85^ These result in a regenerative pheno-type characterized by hyperexcitability and *de novo* ectopic activity.^85,86^ The latter is considered a key driver of pain in people.^4^ Our *in vitro* mechanical trauma model similarly induced expression of canonical injury-associated genes, rapid neurite regeneration and hyperexcitability. The finding that axotomy (in the absence of iMacs) induced a low level of spontaneous activity is consistent with what has been observed in *ex vivo* recordings of rodent sensory neurons from experimental nerve injury models^60^ and human DRG neurons derived from individuals with neuropathic pain^87^ (both situations with limited macrophage presence), suggesting that at least some of the disease features of a damaged sensory neuron are cell intrinsic.

Macrophages have been proposed as a source of pro-inflammatory mediators following nerve injury,^73,88^ some of which have been shown to sensitize sensory neurons in isolation.^89–91^ However, we believe that our findings are the first to demonstrate the capacity of macrophages to directly amplify damaged sensory neuron hyperexcitability, in mouse or human. The finding that iMacs did not impact passive membrane properties or evoked firing properties, but did increase spontaneous (i.e. non-evoked) firing frequency, could have implications for our understanding of the cell type’s role in nerve injury-induced pain.

Experimental depletion of macrophage populations reduces evoked hypersensitivities in preclinical models of nerve injury,^6^ but behavioural readouts of spontaneous pain have not been examined. Both stimulus-evoked and paroxysmal pain scores of Morton’s neuroma patients correlate with a greater presence of macrophage populations in nerve tissue.^7^ Spontaneous activity of sensory neurons is often linked to spontaneous pain,^4^ and so considering our data, and that of Sandy-Hindmarch *et al*.,^7^ it may be prudent to re-evaluate the preclinical models to assess the role of macrophages in maintaining spontaneous pain. If our hypothesis is correct, then it would highlight disrupting pathological macrophage–sensory neuron signalling as an attractive approach to abrogate a major clinical complaint of people living with neuropathic pain.^92^

### Future opportunities

The relative contribution of nerve- and DRG-associated macro-phages to neuropathic pain following nerve injury is contested^5,6^ and we did not seek to model one over the other in this study. While some data we present suggest better alignment of our *in vitro* system with the injury response seen in nerves *in vivo*, we are very hesitant to ascribe this level of specificity to the system. The current 2D co-culture model includes iMacs associating with iSN soma and neurites and will also lack other important tissue-specific signals. Future work, which could include microfluidic compartmentalization and addition of further cell types (e.g. Schwann cells, satellite glial cells, etc.), may help to better model heterogeneity of sensory neuron-associated macrophage populations.

Our model offers a new platform to study sensory neuron–macrophage cross talk, which is known to be important for a myriad of biological processes beyond pathological pain, including normal mechano- and thermo-transduction,^93^ wound repair and fibrosis,^94,95^ and defence against infection.^96,97^ In nerve regeneration, macrophages are classically considered pro-regenerative due to their actions of debris clearance and signalling to Schwann cells.^12,32,98,99^ A study by Hakim and colleagues^100^ has recently described a role for nerve-associated macrophages in protecting against sensory axon loss in a mouse model of diabetic peripheral neuropathy. Of interest, this study identified Galectin-3 upregulation as a key mediator of this role.^100^ iMacs in our co-culture were highly efficient at clearing neuronal debris following damage and neurite regeneration appeared qualitatively changed, with neurites aligning into dense tracts. These observations are consistent with a recent study that demonstrated the ability of iPSC-derived macro-phages to increase naïve neuron neurite growth and induce the formation of nerve bundle-like structures.^101^ While we did not specifically address the contribution of iMacs to neuroprotection and neurite regeneration, this would be a fruitful avenue of investigation.

In summary, we present a humanized co-culture model to study the interactions of sensory neurons and macrophages. We have used this model to show a direct action of macrophages in sensitizing the electrical activity of damaged sensory neurons and to identify candidate signalling pathways that may be amenable to therapeutic disruption.

## Supplementary Material

Supplementary material is available at Brain online.

Supplementary Data

## Data Availability

All raw and processed sequencing files, including Seurat objects, can be obtained from the Gene Expression Omnibus (GEO) under accession GSE298325. All other data and materials will be made available upon reasonable request to the corresponding author.
