# Supplementary material for: Macrophages amplify spontaneous activity of damaged sensory neurons in a human co-culture model of neuropathic pain
Source: Brain. Author manuscript; Available in PMC 2026 Nov 4. (PMC7619696; doi:10.1093/brain/awag088)
Supplement: Supplementary file 1 [file EMS221425-supplement-Supplementary_Data.zip › brain-2025-01904-File007.pdf]

Supplementary figures

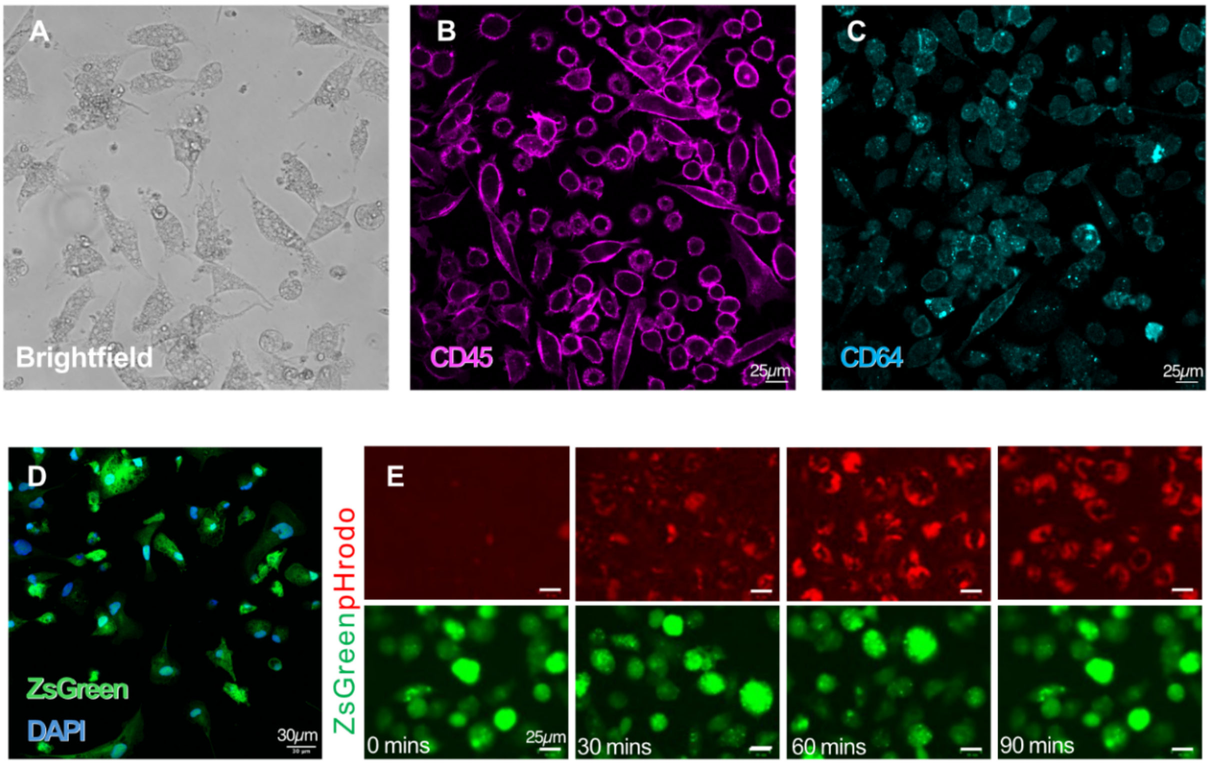

Supplementary figure 1. iMac generation

(A-C) Representative brightfield and fluorescent images of macrophage-like cells (iMacs). Cells stain positive for the hematopoietic cell markers CD45 and CD64. **(D)** Representative image of iMacs produced from the ZsGreen knock-in line. **(E)** Time lapse of ZsGreen-derived iMacs incubated with pHrodo Red E. coli BioParticles, which fluoresce in acidic environments (such as lysosomes). The gradual increase in pHrodo signal indicates iMacs are competent phagocytes.

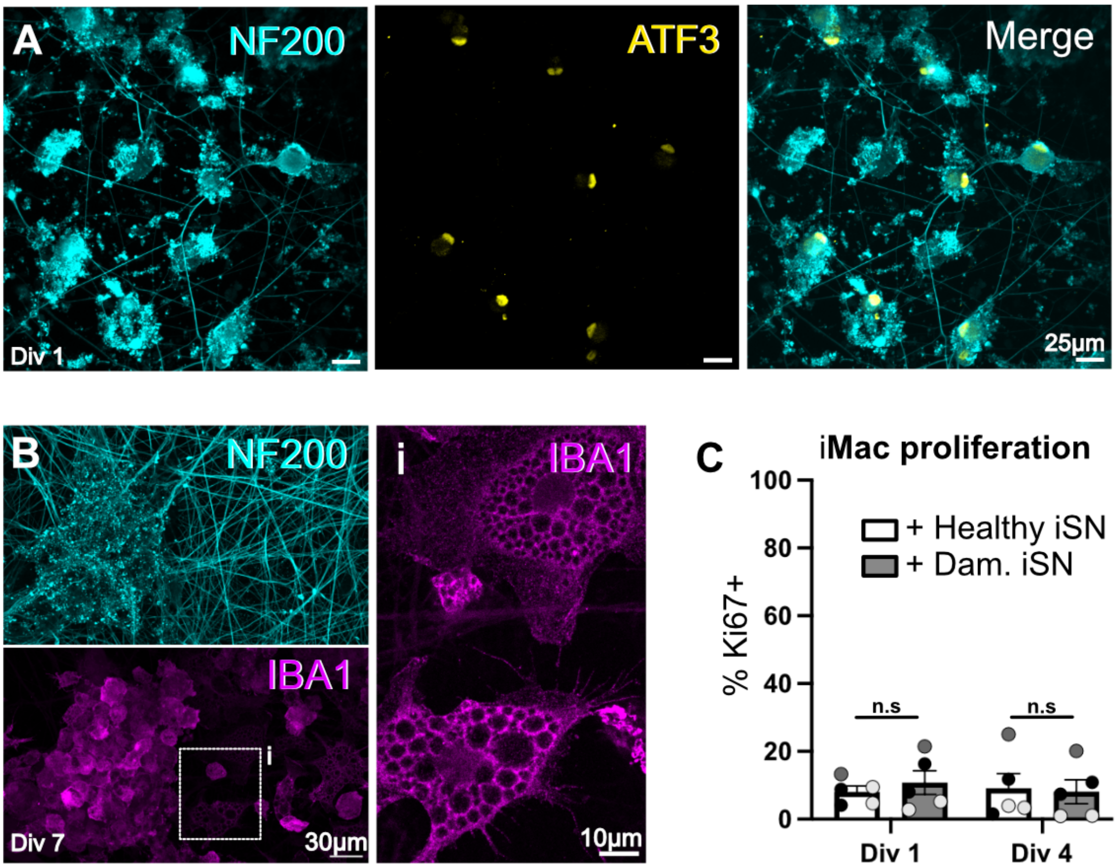

Supplementary figure 2. Co-culture of iMacs with damaged iSNs

(A) iSNs 24hrs after axotomy and replating, illustrating expression of the canonical injury marker, ATF3, and evidence of rapid neurite regeneration. **(B)** iMacs co-cultured with damaged iSNs for 7 days showing the majority of cells clustering to soma of damaged iSNs. Highlighted ROI (**i**) illustrates the large, flattened and often highly vacuolated morphology exhbited by iMacs within regenerating neurite network. Note, B is an orthogonal projection through the whole z-stack of the culture, whereas Bi is an orthogonal projection of only the thinner plane of the flattened iMacs. (**C**) Proportion of iMacs staining positive for the proliferation marker, Ki67, at acute timepoints following co-culture. Data represent mean ± SEM, with values for individual differentiations also shown (n=5, from 3 independent iPSC lines: *black circles - SBAD2, grey circles - SBAD3 and white circles - ZsGreen*). Two-way ANOVA followed by Sidak's multiple comparison test. N.s $P>0.05$.

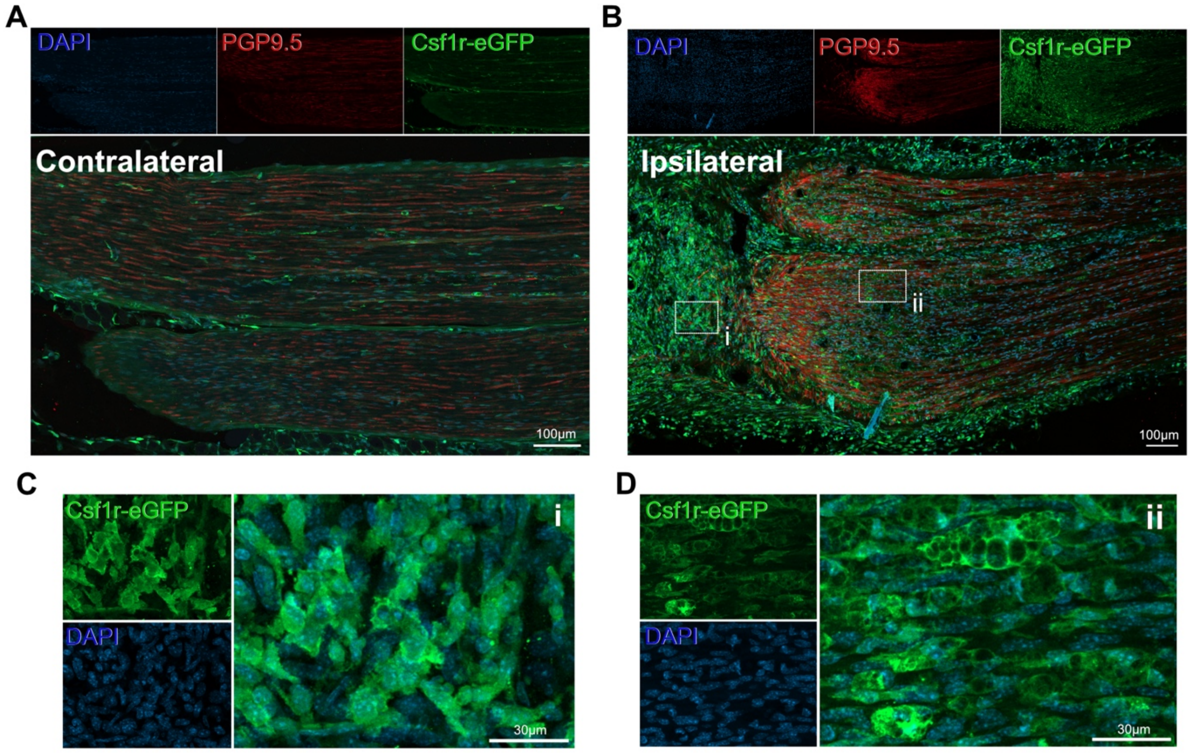

Supplementary figure 3. Nerve-macrophage morphology

Overview of contralateral (**A**) and ipsilateral (**B**) sciatic nerve from Csf1r-eGFP reporter mice 14 days following spared nerve injury (SNI). Ipsilateral nerve is characterised by high numbers of labelled cells, exhibiting different morphologies along the nerve stump. (**C**) Magnified view of cell clusters at the presumed site of nerve transection (**Bi**), some cells of which have a rounded morphology. (**D**) Magnified view of vacuolated cell morphology observed proximal to the site of transection.

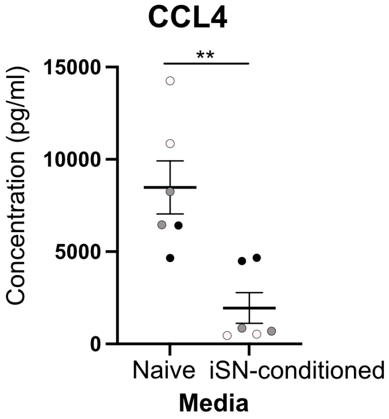

Supplementary Figure 4. Soluble mediators from iSNs supress secretion of CCL4

CCL4 profiling of supernatant samples taken from iMac monocultures. Media was added to mature iSN cultures or blank wells for 3 days before removal and addition to iMacs. Data represent mean ± SEM, with values for individual differentiations also shown (n=6, from 3 independent iPSC lines: *black circles - SBAD2, grey circles - SBAD3 and white circles - ZsGreen*). Mann-Whitney test. $^{**}P<0.01$,

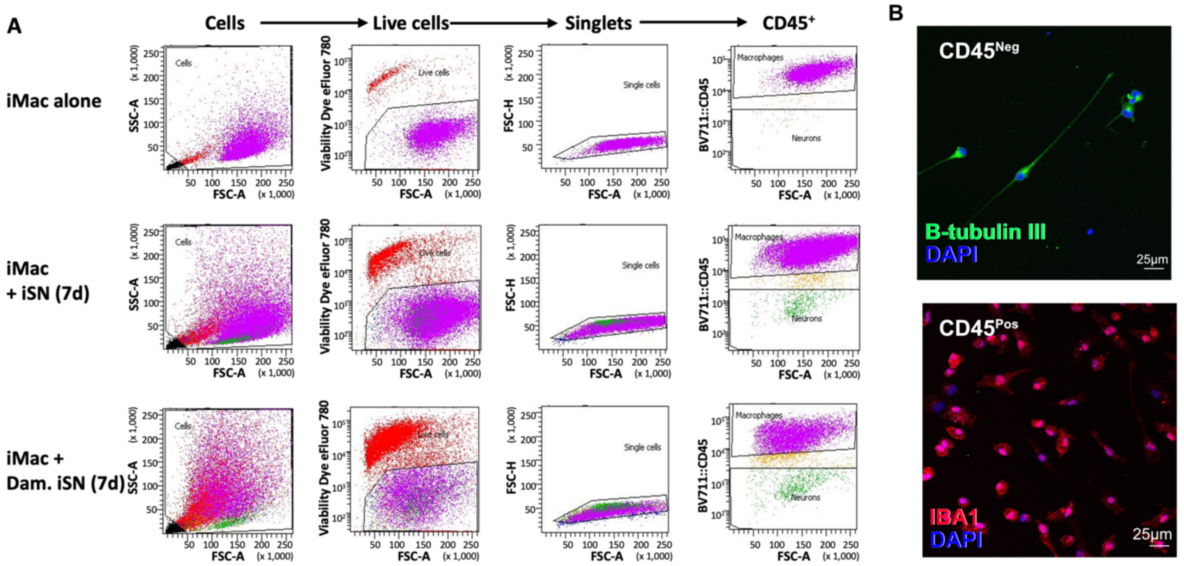

Supplementary figure 5. FACS gating strategy for iMac samples collected for scRNA-seq. (A) Samples containing iMacs cultured alone or with healthy or damaged iSNs for 7 days were gated based on forward (FSC) and side scatter (SSC) to enrich events for cells, live cells (i.e. those which were Viability eFluor™ 780 negative), singlets, and CD45 positive events. Neurons were not routinely collected. **(B)** Representative image of cells taken from CD45 negative and positive FACS gates during a pilot experiment, confirming appropriate capture of the two cell types.

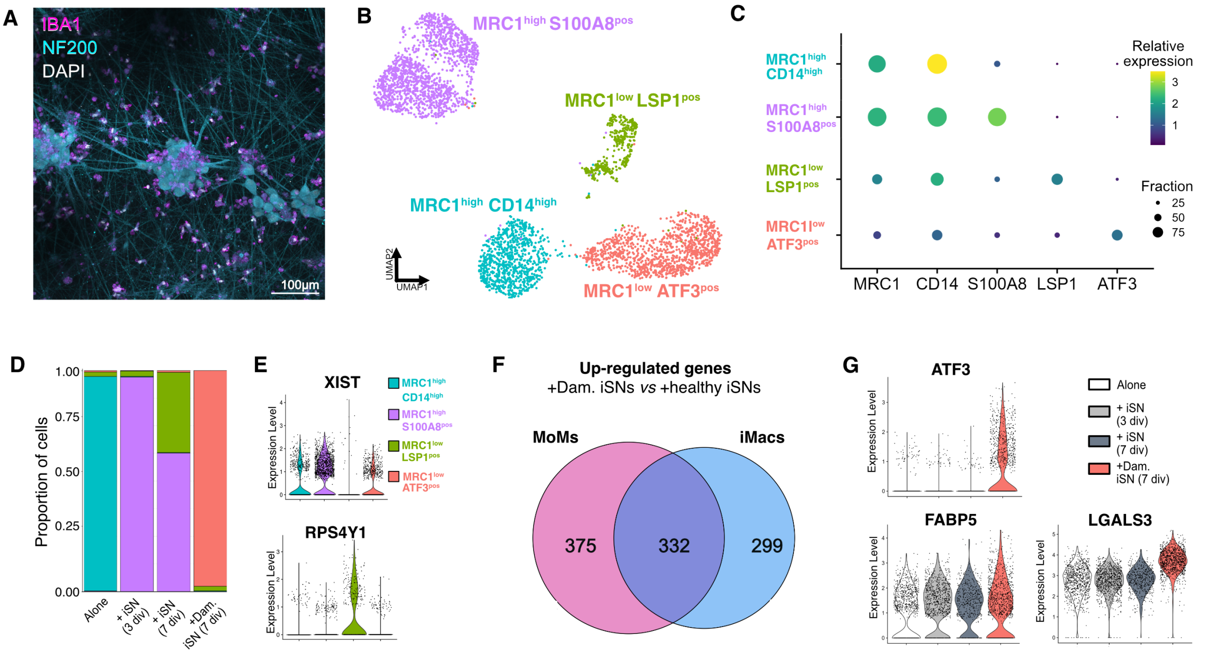

Supplementary figure 6. scRNA-seq analysis of MoMs

(A) Representative image of monocyte-derived macrophages (MoMs) co-cultured with iSNs for 7 days under conditions identical to iMac co-culture. MoMs stain positive for the macrophage marker IBA1. **(B)** UMAP projection of MoMs pooled from 4 conditions; cultured alone (895 cells) and co-cultured with healthy iSNs for 3 (980 cells) and 7 (854 cells) days, or with injured iSNs for 7 days (1113 cells) (Note, n=1 differentiation and 1 donor). Four distinct clusters can be seen. **(C)** Dot plot showing the relative expression of markers used to define clusters. **(D)** Proportion of MoMs from different conditions corresponding to each of the four clusters. Most clusters consist of MoMs from one condition, similarly to iMacs. **(E)** We noted that unlike other clusters, the MRC1^{Low}LSP1^{pos} cluster had low expression of the female-biased gene XIST and enriched expression of the Y-linked gene RPS4Y1, leading to a reasonable suspicion that the female donor derived sample was partially (<10% cells) contaminated with male donor-derived MOMs concurrently in culture. We therefore excluded the MRC1^{Low}LSP1^{pos} cluster from subsequent analysis. **(F)** Venn diagram of genes upregulated in either iMacs or MoMs when co-cultured with healthy *vs* injured iSNs (FDR < 0.05 and $\log_2$FC > 0.5), showing an overlap of approximately 50%. This is far greater than expected by chance: hypergeometric p value = 3.289669e-238. **(G)** Violin plots of three injury-associated genes found to be upregulated in iMacs co-cultured with injured iSNs. ATF3 and LGALS3 are also upregulated in MoMs cultured with damaged iSNs, however no difference is observed in FABP5 expression.

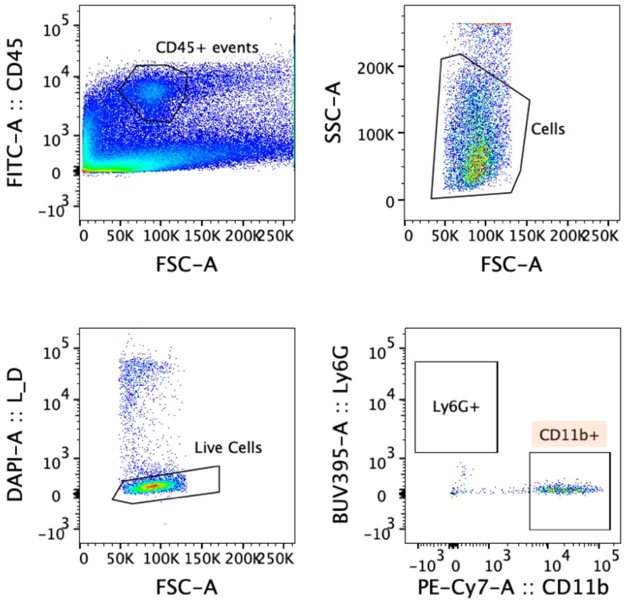

Supplementary figure 7. FACS gating strategy for samples taken at 1-week post-sciatic nerve injury

Data shown here derive from a single female PSNL mouse. CD45+ events were selected, followed by events that are likely cells based on forward (FSC) and side scatter (SSC), single cells based on the area (SSC-A) and width (SSC-W) of the side scatter, and live cells, i.e. those which were DAPI negative. After this, we sorted cells which were negative for the neutrophil marker Ly6G, but positive for the myeloid cell marker CD11b. All gates were placed on fluorescence minus one controls. At day 7 post nerve injury, we were not able to observe neutrophils, in keeping with what we have observed before in the PSNL model, where neutrophil infiltration appears to peak at day 1 post injury (Liang et al., Pain, 2020, 10.1097/j.pain.0000000000001914).

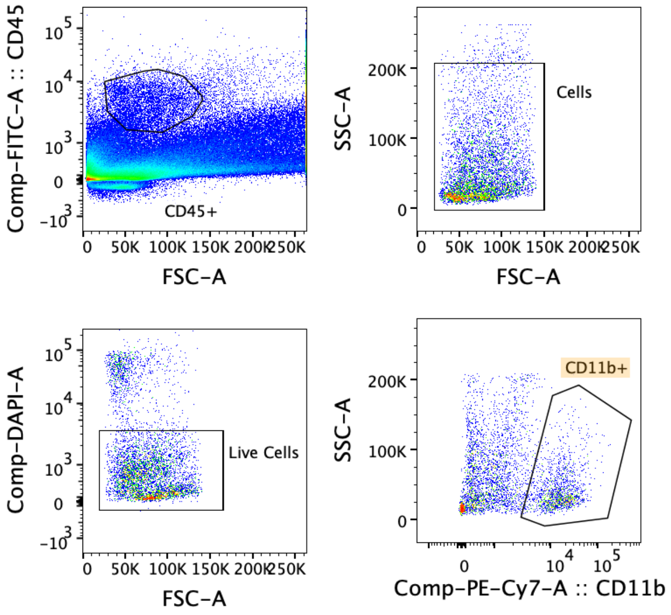

Supplementary figure 8. FACS gating strategy for samples taken at 15-weeks post-sciatic nerve injury.

Data shown here derive from a single female PSNL mouse. CD45+ events were selected, followed by events that are likely cells based on forward (FSC) and side scatter (SSC), single cells based on the area (SSC-A) and width (SSC-W) of the side scatter, and live cells, i.e. those which were DAPI negative. After this, we sorted cells which were positive for the myeloid cell marker CD11b All gates were placed on fluorescence minus one controls.

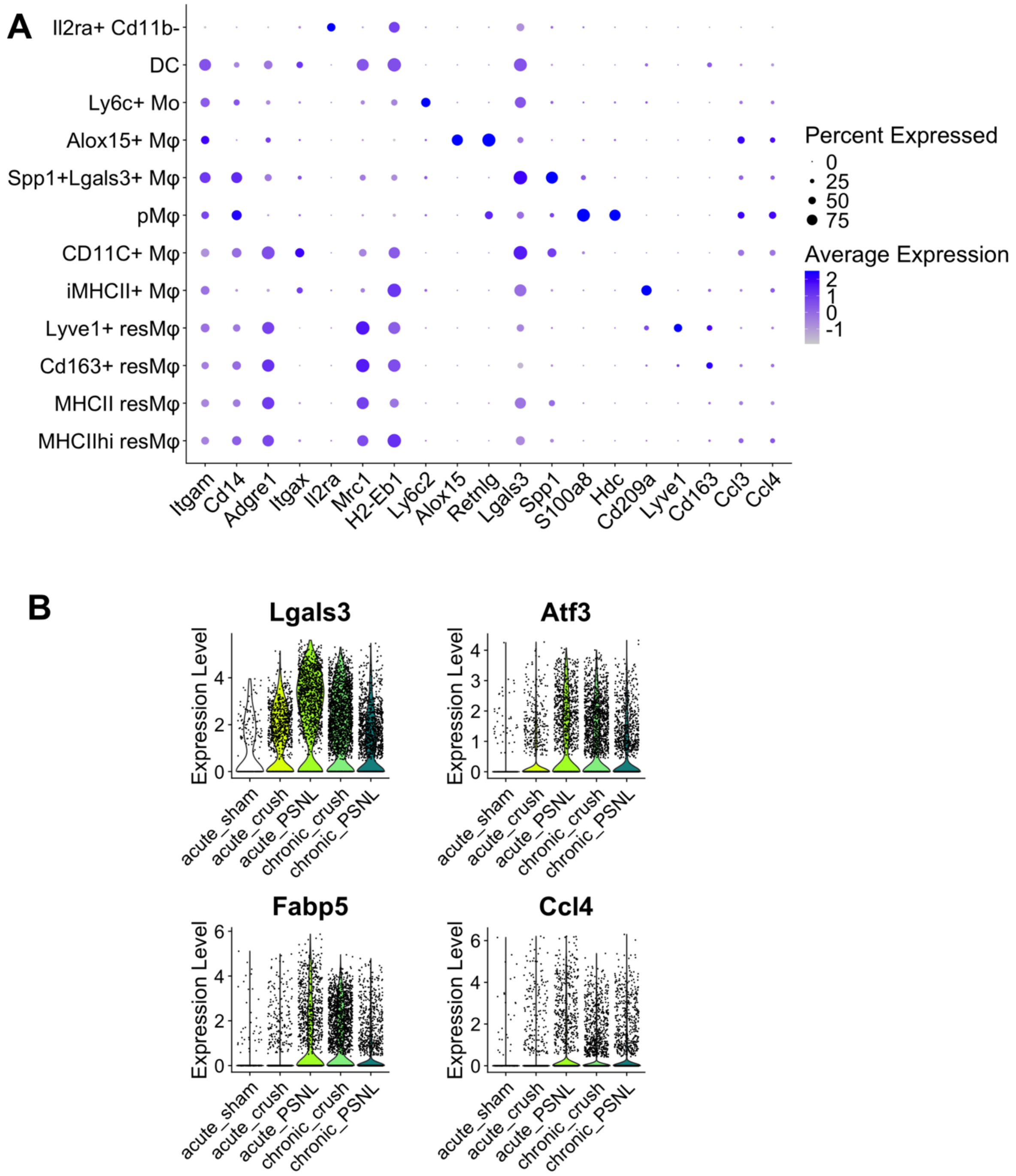

Supplementary figure 9: Mouse scRNA-seq data reveal that equivalent marker genes can be detected *in vivo* to those observed in our human iMac model

(A) Plots showing the expression level of marker genes in different cell clusters observed after sequencing mouse myeloid cells obtained from sciatic nerve either after sham surgery or 1-week or 15-weeks after nerve damage. Mφ = macrophages, p = phagocytic, i = injury-induced, Mo = monocytes, DC = dendritic cells and res = resident. **(B)** Violin plots of Lgals3, Atf3, Fabp5 and Ccl4 expression in the different injury conditions: samples taken one week after

sham, crush or PSNL surgery (acute) vs. samples taken 15 weeks after crush or PSNL surgery (chronic).

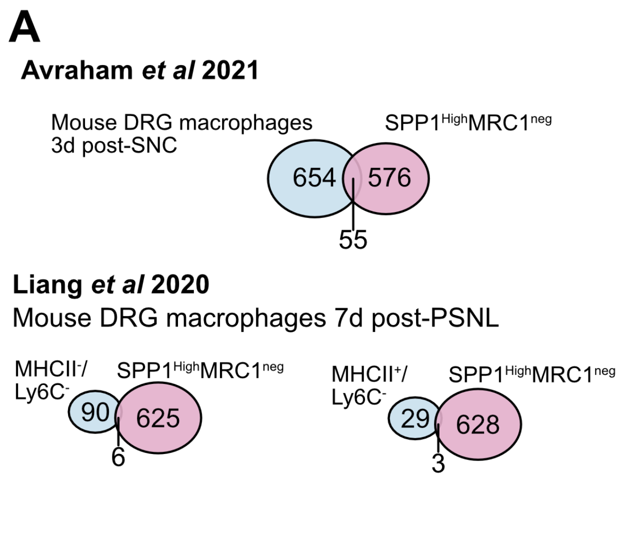

Supplementary figure 10. Comparison of iMac clusters to publicly available murine DRG macrophage and human nerve datasets

(**A**) Venn diagram of genes upregulated in the injury-associated iMac cluster (SPP1^{High}MRC1^{neg}) and two available datasets of DRG macrophages following acute sciatic nerve injury, indicating total number of genes (as defined by the study authors) and overlap with SPP1^{High}MRC1^{neg} cluster. Neither dataset exhibited significantly greater overlap than might be predicted by chance (hypergeometric *p* value>0.05). (**D**) Violin plot showing the Macro18 module score for each cluster in our iMac dataset. The score was computed based on the top 300 DEGs defining the Macro18 cluster and shows the greatest enrichment in our injury-induced SPP1^{High}MRC1^{neg} iMac cluster.

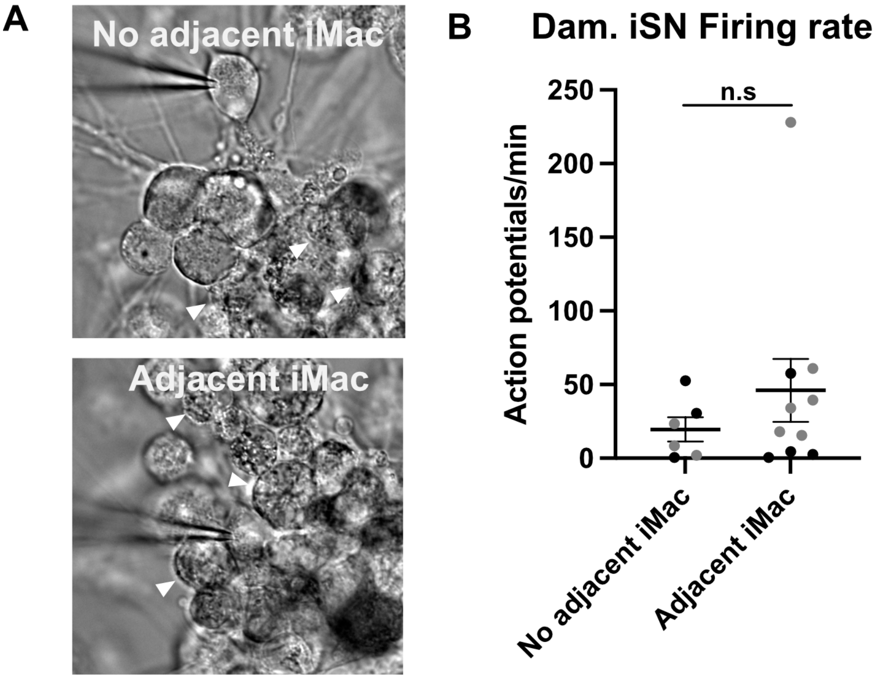

Supplementary Figure 11. iMacs augment iSN spontaneous activity without direct contact with neuronal soma.

(A) Patch-clamp recordings were taken from damaged iSNs in co-culture with iMacs for 7 days following injury. Representative brightfield images of iSNs during recording, showing examples of those classified as adjacent, or not adjacent to at least one iMac. White arrows highlight example iMacs in the images. (**B)** Action potential firing frequency of spontaneously active damaged iSNs. All data represent mean ± SEM with values for individual iSNs also shown (derived from 2 independent iPSC lines: *Black circles SBAD3, grey circles- ZsGreen and a total of* 25 *(no adjacent iMac) and 31 (adjacent iMac) recordings)*. Mann-Whitney test. N.s *P*>0.05.

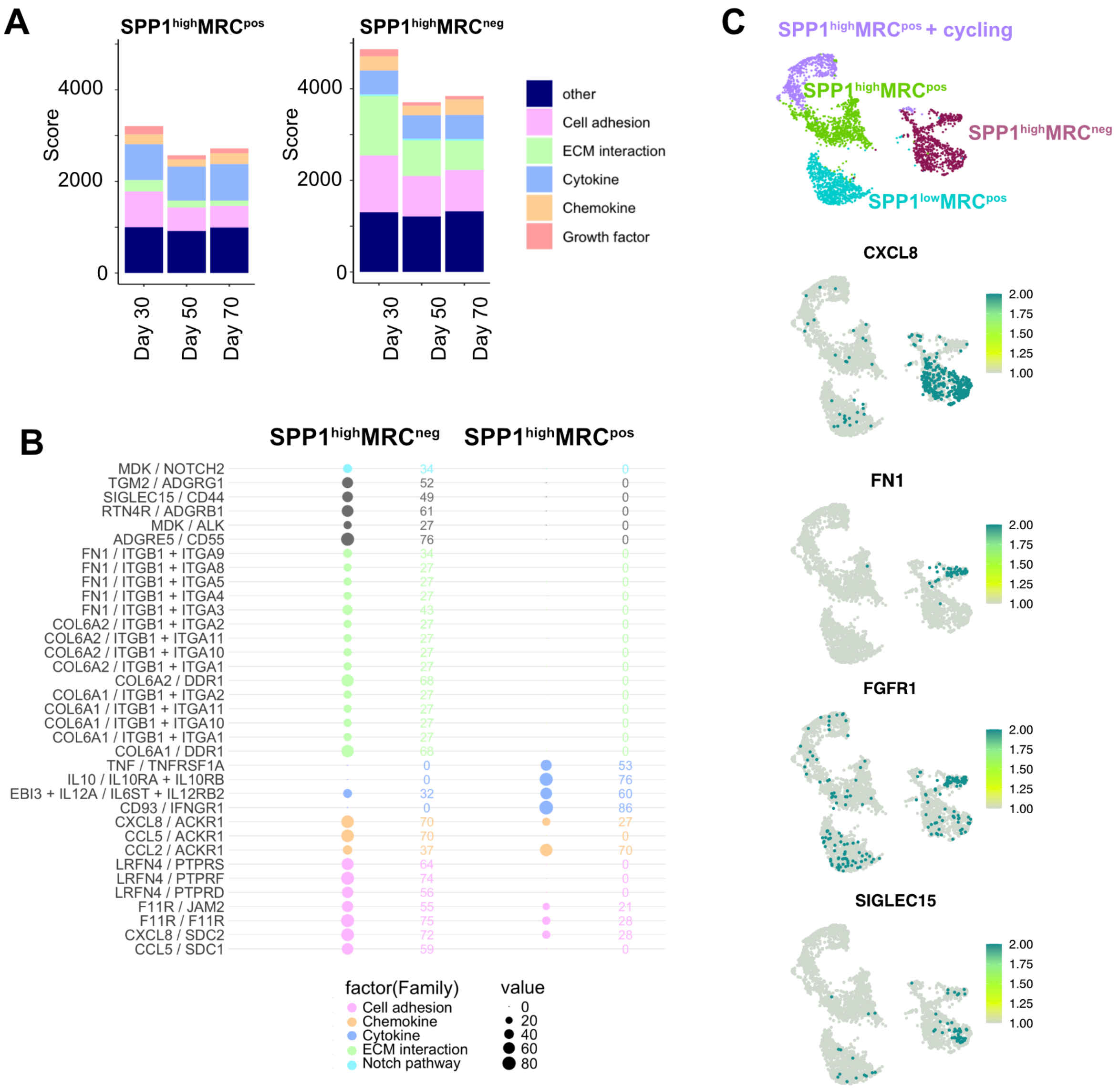

Supplementary Figure 12. Ligand receptor interactions.

(A) Communication scores for ligand-receptor interactions between iSNs differentiated for different lengths of time and SPP1^{high}MRC^{pos} (which predominate in the healthy iSN condition) and SPP1^{high}MRC^{neg} iMacs (which predominate in the damaged iSN condition). iSN RNA-seq data originally published by Li et al., Pain, 2025, 10.1097/j.pain.0000000000003512; day 50 corresponds to the age of the iSNs grown in our functional experiments. Scores are plotted for different ligand-receptor families with higher scores representing a greater number of matching ligand-receptor pairs expressed by the sender (iMac population) and receiver (iSNs). **(B)** Top differential ligand receptor pairings comparing the two iMac populations. The colours denote the function of each ligand-receptor pair, the size of the circles and corresponding numbers represent the communication scores. **(C)** UMAP feature plots of ligands with enriched

expression in $SPP1^{high}MRC^{neg}$ for which there is prior data implicating either the ligand or a cognate receptor in sensory neuron excitability.

**Parameter**	**Injury**	**iMac presence**	**Interaction (Injury x iMac presence)**
**Resting membrane potential**	$F_{1, 123} = 9.226$ *P*=0.0029	$F_{1, 123} = 7.182$ *P*=0.0084	$F_{1, 123} = 0.3720$ *P*=0.5430
**Input resistance**	$F_{1, 121} = 22.78$ *P*<0.0001	$F_{1, 121} = 0.1844$ *P*=0.6684	$F_{1, 121} = 0.5834$ *P*=0.4465
**Capacitance**	$F_{1, 115} = 97.94$ *P*<0.0001	$F_{1, 115} = 4.143$ P=0.0441	$F_{1, 115} = 3.354$ *P*=0.0696
**Rheobase**	$F_{1, 119} = 58.45$ *P*<0.0001	$F_{1, 119} = 0.5362$ *P*=0.4654	$F_{1, 119} = 2.348$ *P*=0.1281

Supplementary Table S1

Two-way ANOVA degrees of freedom and *P* values for analyses of the effects of injury (healthy *vs* damaged iSNs) and/or presence of iMacs (related to Figure 5).

Supplementary Methods

iPSC culture and maintenance

iPSCs were maintained in mTesR Plus media (Stem Cell Technologies, #100-0276) on Matrigel (Corning, #354234) coated dishes. When confluent, cells were passaged as aggregates with ReLeSR (Stem Cell Technologies, #05872). When cells were required to be plated as single cells prior to differentiation, they were instead passaged by Accutase treatment (ThermoFisher, #A1110501), with plating medium supplemented with CEPT cocktail (chroman 1, emricasan, trans-ISRIB; Tocris, #59222C, #M1028 and #I8896 respectively).

iPSC-derived sensory neuron (iSN) generation

iPSCs were differentiated to iSNs as per[2], with only minor modifications. Cells were plated on Matrigel treated plates and expanded in mTESR Plus medium until 40-50% confluent, after which they were switched to KSR base medium, which was composed of Knockout-DMEM (ThermoFisher, #10829018), 15% knockout-serum replacement (ThermoFisher, #10828028), 1% Glutamax (ThermoFisher, #35050038) 1% nonessential amino acids (ThermoFisher, #11140050) and 100 μM β-mercaptoethanol (ThermoFisher, #31350-010). This timepoint is denoted as day 0. Neural induction was initiated by adding SMAD inhibitors SB431542 (10 μM , Tocris, #1614) and LDN-193189 (500 nM, Tocris, #8150) until day 5. Three additional small molecules were introduced on day 3; CHIR99021 (3 μM, Tocris, #4423), SU5402 (10 μM, Stratech, #3300) and DAPT (10 μM, Tocris, #2634). The culture medium was gradually transitioned to N2/B27 medium (Neurobasal medium (ThermoFisher, #21103049), 2% B27 supplement (ThermoFisher, #12587010), 1% N2 supplement (ThermoFisher, #17502048), 1% Glutamax) in 25% increments from day 4. Cells were passaged with Accutase and replated onto glass coverslips at day 12 of the differentiation in N2/B27 medium supplemented with four recombinant growth factors at 25ng/ml (BDNF (Peprotech, #450-02), NT3 (Peprotech, #450-03), NGF (Peprotech, #450-01) and GDNF (Peprotech #450-10)). CHIR90221 was included for a further 4 days. Cytosine arabinoside (4 μM, ThermoFisher, #15244774) was added to cells for 18hrs between days 14 and 20 to remove contamination with dividing cells. From day 25 onwards, cells were maintained in N2/B27 Plus medium (Neurobasal Plus medium (ThermoFisher, #A3582901), 2% B27 Plus supplement (Thermofisher, #A3582801),

1% N2 supplement, 1% Glutamax supplemented with 25ng/ml BDNF, GDNF, NT3 and NGF, and Matrigel (1:500). Medium was 50% refreshed 3 times per week and cells used for experiments >45 div. For fluorescent labelling of neurons, AAV.PHP.s.hSyn.mCherry (University of Zurich Viral Vector Facility) was added one week prior to experiments at an MOI of 10^5.

iMac generation

iPSCs were differentiated to iMacs as per[3], with only minor modifications. Single-cell suspensions of $4x10^6$ iPSC cells were seeded into an Aggrewell 800 well (STEMCELL Technologies, #34825) to form embryoid bodies (EBs). These cells were maintained in mTesR Plus media supplemented with 50 ng/mL BMP4 (ThermoFisher, #PHC9534), 50 ng/mL VEGF (ThermoFisher, #PHC9391), and 20 ng/mL SCF (ThermoFisher, #PHC2111). CEPT cocktail was included in the medium on the day of seeding. Media was 75% refreshed daily for 6 days before EBs were plated on Matrigel-treated T25/75 flasks at an approxmate density of one EB/cm^2. EBs were maintained in flasks in X-VIVO15 media (Lonza, #02-060Q) supplemented with 100ng/ml M-CSF (ThermoFisher, #PHC9501), 25ng/ml IL-3 (ThermoFisher, #PHC0031), 1% Glutamax, 0.055mM β-mercaptoethanol and 1% antibiotic-antimycotic (anti-anti, ThermoFisher, #15240096). Media was 50% refreshed weekly until the emergence of iMac precursors in the supernatant at which point 100% media changes were performed twice weekly. iMac precursors were harvested from the supernatant and plated for 7 days in X-VIVO15 media supplemented with 100ng/ml M-CSF, 1% Glutamax, and 1% anti-anti, to promote final differentiation. To confirm phagocytic activity, pHrodo Red S. aureus BioParticles (ThermoFisher, #P35367) were added to iMacs at 50 μg/ml and fluorescence imaged every 30mins on a Zoe Fluorescent Cell Imager (BioRad).

Monocyte-derived macrophages (MoMs)

30ml of peripheral blood was collected from a healthy 47yr-old female, in accordance with the University of Glasgow's MVLS Ethics Committee (Project No: 2012073), and with written consent. PBMCs were isolated following density gradient centrifugation with Histopaque-1077 (Sigma Aldrich, #10771) and $CD14^{Pos}$ cells were isolated using CD14 microbeads and AutoMACSPro (Miltenyi BioTec, #130-050-201), according to the manufacturer's protocol.

These cells were plated into six-well culture plates and differentiated into monocyte-derived macrophages by 7 days of culture in RPMI 1640 medium (ThermoFisher, #31870-025) containing 10% foetal bovine serum (ThermoFisher, #10439001), 1% anti-anti and 50ng/ml M-CSF (complete RPMI media). Media was replenished on day 3 and cells increased their adherence throughout the 7 days.

Single cell RNA sequencing (scRNA-seq) of iMacs/MoMs

Sorted cells were manually counted with a haemocytometer and 2,500 cells per condition were mixed for a total of 20,000 cells. Cells were loaded onto a Chromium Controller (10X Genomics) for single-cell partitioning, using Chromium Next GEM Chip G Single Cell Kit (10X Genomics #PN-1000120), and single-cell 3' Reagent Kits v3.1 (10X Genomics #PN-1000121). A library consisting of iMacs and MoMs was sequenced on an Illumina Nextseq 500 according to the manufacturer's protocol, at the Glasgow Polyomics facility at a depth of 20,000 reads/cell. Reads were mapped to the human genome (GRCh38-3.0.0) using 10X CellRanger (version 7.0.0).

scRNA-seq analysis of iMacs/MoMs

Analysis was carried out using the Seurat package (version 5.2.1) in RStudio (version 4.4.2). Briefly, the data were loaded using the Read10X() and CreateSeuratObject() functions. Prior to downstream analysis, the library was filtered to only retain cells with > 200 and < 5000 unique genes as well as cells in which < 10% of the counts were from mitochondrial genes for downstream analysis. Counts were subsequently scaled to 10,000 transcripts per cell using the NormalizeData() function and highly variable genes were identified using FindVariableFeatures(). The data were then scaled to equalise variance amongst genes and dimensionality reduction was performed using the RunPCA() function. A shared K-nearest neighbour (KNN) graph was constructed to identify the nearest neighbours for each cell based on the first 20 principal components (PCs) using the FindNeighbours() function and clustering was performed using FindCusters() at a resolution of 0.2. For visualisation, uniform manifold approximation and projection (UMAP) were generated with coordinates calculated from the first 20 PCs using the RunUMAP() function.

Prior to further analysis, samples were deconvoluted by the addition of HTO information using cellhashR (v 1.0.3) (Table 1). Cells with no hashtag information and doublets were removed, and the UMAP was re-generated using the pipeline described above. The library was further divided into two distinct datasets, iMacs and MoMs, each containing macrophages from four conditions. These were analysed separately following the steps described previously. Clustering was performed at a resolution for 0.1 for the iMac and 0.15 for the MoM datasets. Differential expression analysis was carried out using the FindAllMarkers() function (min.pct=0.25, test="MAST") and clusters were labelled based on the top 15 differentially expressed genes (FDR < 0.05).

To examine the overlap between DEGs found in our injury-induced iMac cluster and mouse DRG-resident macrophages following nerve injury, we used data from two publicly available datasets. Transcriptomic data from Avraham *et al.* was accessed via the NCBI GEO database (GSE158892) and only uninjured and sciatic nerve crush L4 DRG samples were included. For the Liang *et al.* dataset, significantly upregulated DEGs in the MHCII^{-}/Ly6C^{-} and MHCII^{+}/Ly6C^{-} populations were obtained from Supplementary Table 5 (GSE139150). Lastly, a comparison of iMacs to nerve resident macrophages from patients with polyneuropathy (Heming *et al.*) was performed using the UCell package. An injury signature was computed from the top 300 differentially expressed genes (log2FC > 0.5 and p_val_adj < 0.05) provided in Supplementary Data 5 of the publication, and human orthologues of the mouse genes were identified in the iMac dataset. All raw and processed sequencing files, including Seurat objects, can be obtained from the Gene Expression Omnibus (GEO) under accession GSE298325. The full analysis code is provided in Supplementary RNotebook 1.

**HTO**	**Condition**
HTO1	iMacs alone
HTO2	MoMs alone
HTO3	iMacs + healthy sensory neurons (3 days)
HTO5	MoMs + healthy sensory neurons (3 days)
HTO6	iMacs + healthy sensory neurons (7 days)
HTO8	MoMs + healthy sensory neurons (7 days)
HTO9	iMacs + damaged sensory neurons (7 days)

HTO10	MoMs + damaged sensory neurons (7 days)

Table 1. Hashtag oligo (HTO) barcodes used in CITE-seq multiplexing and their corresponding conditions.

Surgical nerve damage in mice

For all nerve injury procedures, animals were anaesthetised under 2% isoflurane and pre-operatively administered 0.1mg/kg of the analgesic buprenorphine subcutaneously (s.c). After shaving the fur covering the left hip and leg region, the sciatic nerve was exposed via blunt dissection. For PSNL, roughly half of the nerve was tightly ligated with a suture (5.0 Vicryl suture, Ethicon, #W9982). For nerve crush, Watchmaker forceps (Dumont, #0304-5-PO) were placed horizontally around the nerve and pressed tightly for 10 seconds, the forceps were then placed vertically around the same section of nerve and again pressed tightly for 10 seconds. For spared nerve injury (SNI), the common peroneal and tibial nerves were ligated using a 6-0 Vicryl suture (Ethicon, Raritan, NJ), and a 1- to 2-mm piece distal to the suture was removed using spring scissors. For sham surgeries the sciatic nerve was exposed but left untouched. In all cases, the wound was stapled shut with a Reflex 7mm metal wound clip (Fine Science Tools, #12032-07). Mice were transferred to a clean cage and left to recover in the animal unit. Animals were weighed and checked for autotomy daily for at least 3 days following surgery and wound clips were removed within 7 days of surgery.

Nerve dissociation and processing

1 week or 15 weeks after surgery, mice were transcardially perfused with 10 ml PBS to remove leukocytes from circulating blood. Ligated, crushed or uninjured (sham) nerves were dissected and placed into a petri dish containing F12 medium (Gibco, #21765-029). Ligatures were removed with tweezers, if still present, and nerves were cut to 0.5 cm, ensuring equal lengths of nerve either side of the ligation/crush site. Following dissections, samples were transferred into wells of a standard 96-well PCR plate containing 51µl of the following digestion mix: 6.25 mg/ml collagenase type IA (Sigma Aldrich, #C9891), 0.2% Pronase (Millipore, #53702), 0.4% Hyaluronidase (ABNOVA, #P52330) in F12 medium. Spring scissors were used to chop the tissue into small pieces (50x chops, cleaning scissors between samples with 100% ethanol). After this manual dissociation, the plate was incubated on a shaking platform (220 rotations

per minute) at 37°C for 30 minutes. The digestion mix was subsequently removed and replaced with 100µl of fluorescence-activated cell sorting (FACS) buffer: 0.4% BSA (Sigma-Aldrich, #A3983), 15 mM HEPES (Gibco, #15630080), 2mM EDTA (Invitrogen, #15575038) in Hank's Buffered Saline Solution (HBSS, diluted from 10x, Gibco, #14175095). Samples were then further dissociated by pipetting up and down 50x, with the resulting single-cell solution filtered through a 35µm cap of a BD falcon tube with a cell strainer cap (BDBiosciences, # 352235). The plate was centrifuged at 1500 rotations per minute for 5 minutes at 4°C, after which supernatants were removed and resuspended in 50µl of the following staining mixture for 30 minutes at 4°C in a dark fridge: Fc block (BioLegend, #101302, 1:200), FITC-CD45 to stain leukocytes (BioLegend, #147709, 1:1200), PE-Cy7-CD11b to stain myeloid lineage cells (BioLegend, #101215, 1:1200) and, depending on the sample, one of four hashtag antibodies: TotalSeq-B0301-MHCI (BioLegend, #155831), TotalSeq-B0302-MHCI (BioLegend, #155833), TotalSeq-B0303-MHCI (BioLegend, #155835), TotalSeq-B0304-MHCI (BioLegend, #155837). At the acute time point, one week after surgery, the antibody staining mix also contained BUV395-Ly6G to stain neutrophils (BD Bioscience, #563978, 1:300). After staining, cells were centrifuged for 5 minutes at 1,500 rotations per minute at 4°C, after which the cell pellets were resuspended in 300µl FACS buffer and transferred to FACS tubes, adding DAPI to stain for dead cells (Invitrogen, #D1306, 1µl of a 1:2 dilution).

FACS of mouse nerves

Sorting was performed on a BD FACSAria II or BD FACSAria Fusion in the Advanced Cytometry Platform Core Facility of the Guy's and St Thomas' NHS Foundation Trust. Gating strategies are shown in Supp figs 6 and 7. Unstained cells, DAPI single stained cells and single stained control beads (BD™ CompBeads, BD Bioscience, #552845) were used for compensation. Cells were sorted into 1.5ml microcentrifuge tubes containing 4µl of 4% BSA (Sigma-Aldrich, #A3983) in PBS. All tubes were pre-coated for at least 1 hour with the same 4% BSA solution and placed on ice immediately following cell collection. The following samples were collected: for the 1-week time point, CD45+/CD11b+/Ly6G- live single cells from n = 2 sham (2,000 cells each), crush and PSNL mice (8,000 cells each); for the 15-week time point, as many CD45+/CD11b+ live single cells as possible were collected from n = 3 crush and n = 4 PSNL mice. Samples were processed and analysed as detailed in Supplementary materials.

scRNAseq of mouse myeloid cells

After FACS, cells were immediately taken to the Guy's and St Thomas' Genomics Core Facility which was located on the same floor as the Advanced Cytometry Platform. Hashed samples were combined as follows in two separate 10X runs: for the 1-week time point, one 10X well (acute crush) was filled with cells from 1x male sham, 1x male crush, 1x female crush mouse, while the other (acute PSNL) was filled with cells from 1x female sham, 1 male PSNL and 1 female PSNL mouse. A separate 10X run was performed for the 15-week time point, with one well (chronic crush) filled with 2x female crush and 1x male crush sample, and the other well (chronic PSNL) filled with 2x female and 2x male PSNL samples. Libraries were generated using the 10X Genomics Chromium Single Cell 3′ Feature Barcode Library & Gel Bead Kit (v3 Chemistry) according to manufacturer's instructions and sequenced on an Illumina NextSeq 2000 P2 Flow Cell obtaining mean reads per cell of: 24,752 (acute crush well), 35,710 (acute PSNL well), 45,531 (chronic crush well), and 59,220 (chronic PSNL well).

Data analysis of mouse scRNA-seq

Reads were aligned using the 10X Genomics Cell Ranger (version 6.0.0) "cellranger count" pipeline, using the pre-built mouse GRCm38 transcriptome reference and following guidance for "Feature Barcode Analysis" using TotalSeq-B antibodies. The following cell numbers were captured: 1,920 (acute crush), 2,471 (acute PSNL), 2,675 (chronic crush), 2,067 (chronic PSNL). Output files from Cell Ranger were analysed in R Studio (Version 2024.09.1) with Seurat (version 5.1.0)[4]. Within each time point, samples were run on the same 10X chip and sequenced in parallel, minimizing technical batch effects. We therefore first analysed the acute and chronic timepoint data separately. The following cells were excluded to eliminate any empty cells or potential duplets: cells with less than 150 or more than 4,000 (acute) or 4,500 (chronic) unique molecular identifiers (UMIs); cells with more than 20,000 molecules (acute only); cells with more than 10% mitochondrial gene content. Cells negative for a hashtag or positive for more than a single hashtag were also excluded. Cluster identity was assigned based on known marker gene expression. To combine acute and chronic timepoints, three different integration methods were compared in Seurat, with reciprocal PCA deemed to best replicate the clusters that emerged from the individual time-point analyses. See Supplementary RNotebook 2 and its corresponding html file for analysis code and output. For comparison with

the human macrophage scRNAseq data, we used the UCell package[5] to project marker genes for the SPP1^{high} and SPP1^{low} iMac clusters onto the mouse scRNAseq UMAP space. Marker genes were identified using the Seurat FindAllMarker function; to be included in the analysis, they had to expressed in at least 10% of cells in each cluster and had to differ in their expression from the other clusters by at least 20% (SPP1^{low} MRC1^{pos} and mixed SPP1^{high} MRC1^{pos} clusters) or 30% (SPP1^{high} MRC1^{neg}, cycling cell clusters) at adjusted $p < 0.05$. See Supplementary Data Table 4 for the gene lists we obtained and Supplementary RNotebook 2 for the analysis code. We also made a Sankey plot to directly compare mouse and human scRNA-seq clusters. For this, we identified human orthologues of the mouse genes in our data (Supplementary RNotebook 2). Finally, we performed ligand receptor analysis using the ICELLNET package[6], investigating which ligands on our human iMacs would be able to bind receptors present on iSNs. For this, we used previously published RNA sequencing data of iSNs differentiated for 30, 50 and 70 days[7]. Only genes that were deemed expressed on iSNs in this dataset, as well as genes that were expressed in at least 10% of iMac clusters were included in the analysis. See Supplementary RNotebook 3 and its html output for the script that was used. All raw and processed sequencing files, including Seurat objects, can be obtained from the Gene Expression Omnibus (GEO) under accession number GSE298326.

References

1. Liang, Z. *et al.* A transcriptional toolbox for exploring peripheral neuroimmune interactions. *Pain* 161, (2020).
2. Chambers, S. M. *et al.* Combined small-molecule inhibition accelerates developmental timing and converts human pluripotent stem cells into nociceptors. *Nat Biotechnol* 30, 715–720 (2012).
3. Haenseler, W. *et al.* A Highly Efficient Human Pluripotent Stem Cell Microglia Model Displays a Neuronal-Co-culture-Specific Expression Profile and Inflammatory Response. *Stem Cell Reports* 8, 1727–1742 (2017).
4. Hao, Y. *et al.* Dictionary learning for integrative, multimodal and scalable single-cell analysis. *Nat Biotechnol* 42, 293–304 (2024).
5. Andreatta, M. & Carmona, S. J. UCell: Robust and scalable single-cell gene signature scoring. *Comput Struct Biotechnol J* 19, 3796–3798 (2021).
6. Noël, F. *et al.* Dissection of intercellular communication using the transcriptome-based framework ICELLNET. *Nat Commun* 12, 1089 (2021).

7. Li, Y. *et al.* Modelling inflammation-induced peripheral sensitization in a dish—more complex than expected? *Pain* (2025).
